# Natural disaster emergency response from a public policy perspective: a four-party evolutionary game among government, international organizations, healthcare institutions, and enterprises

**DOI:** 10.3389/fpubh.2025.1595034

**Published:** 2025-11-05

**Authors:** Baoling Wu, Tao Zhang, Xi Wang, Jiakai Liang, Mingke Liu, Yifan Zheng, Jiarui Liang, Zhengyu Chen

**Affiliations:** ^1^Faculty of Humanities and Social Sciences, Macao Polytechnic University, Macao, Macao SAR, China; ^2^The Second Affiliated Hospital, Guangzhou Medical University, Guangzhou, Guangdong, China; ^3^Guangzhou Twelfth People's Hospital, Guangzhou, Guangdong, China; ^4^School of Public Health, Guangzhou Medical University, Guangzhou, Guangdong, China; ^5^School of Public Health and Primary Care, The Chinese University of Hong Kong, Hong Kong SAR, China

**Keywords:** natural disasters, emergency response, evolutionary game, public policy, multi-party collaboration

## Abstract

**Objective:**

This study utilizes evolutionary game theory to analyze the collaborative evolutionary mechanisms among governments, international organizations, healthcare institutions, and enterprises in natural disaster emergency response, aiming to explore how public policy can optimize the behavior of each stakeholder.

**Methods:**

A four-party evolutionary game model was constructed to examine strategy interactions and cooperative mechanisms among all parties. Numerical simulations were conducted to verify how key parameters affect the evolutionary outcomes.

**Results:**

The results indicate that government regulatory intensity, intervention strategies of international organizations, the philanthropic orientation of healthcare institutions, and the sense of corporate social responsibility among enterprises significantly influence the efficiency of emergency response. Numerical simulations further show that increasing government penalties, reducing international organizations’ dependency losses, improving the resource utilization efficiency of healthcare institutions, and raising both the cost of non-compliance and the market trust benefits for enterprises can encourage stakeholders to adopt more cooperative strategies that serve the public interest.

**Conclusion:**

This study reveals the “double-edged sword effect” of government regulation, the “time window effect” of international organizational intervention, the “multiplier effect” of resource efficiency in healthcare institutions, and the “trust-benefit mechanism” of corporate social responsibility, offering new insights for optimizing public policy.

## 1 Introduction

With the intensification of global climate change and geological activity, the frequency and severity of natural disasters—such as earthquakes, floods, hurricanes, and droughts—continue to rise. These events not only threaten human life but also exert long-term impacts on global economic and social systems. According to the *Global Disaster Report 2020* released by the United Nations Office for Disaster Risk Reduction (UNDRR) (1), more than 7,348 major natural disasters occurred worldwide between 2000 and 2019—approximately a 75% increase compared with the 4,212 disasters recorded from 1980 to 1999. Over this period, disasters caused about 1.23 million deaths, affected 4.2 billion people, and resulted in economic losses totaling 2.97 trillion USD. The World Economic Forum’s *Global Risk Report 2024* (2) further identifies extreme weather events as the most critical global risk over the next decade, closely linked to biodiversity loss and ecosystem collapse, with potentially irreversible consequences for the planet’s environment.

The sudden onset of natural disasters and their wide-ranging effects on society, the economy, and livelihoods necessitate effective collaboration among multiple stakeholders—including governments, international organizations, healthcare institutions, and enterprises—throughout the phases of emergency response and post-disaster recovery. However, the interaction of stakeholder interests, competition for resources, strategic choices, and collaborative mechanisms ultimately determines the efficiency and effectiveness of disaster response efforts (3).

Public policy plays a crucial role in coordinating stakeholder actions and optimizing resource allocation. In the context of natural disaster emergency response, scientific policy formulation and effective policy implementation can foster collaboration among all parties, facilitating the rapid mobilization and efficient utilization of emergency resources (4, 5). Evolutionary game theory offers a powerful framework for understanding and analyzing the interactions among multiple stakeholders in complex, dynamic settings (6, 7). By employing an evolutionary game model, researchers can analyze how governments, international organizations, healthcare institutions, and enterprises adjust their strategies through repeated interactions—revealing how cooperation emerges, stabilizes, or fails. Such insights offer theoretical guidance for designing more effective public policies.

Drawing on evolutionary game theory, this study focuses on the collaborative evolutionary processes among governments, international organizations, healthcare institutions, and enterprises within the realm of natural disaster emergency response, and examines the role and impact of public policy in this context. We propose a four-party game model to analyze the strategic interactions and cooperative mechanisms among these stakeholders, and explore how public policy can optimize their behavior to enhance both the efficiency and effectiveness of disaster emergency response.

## 2 Literature review

### 2.1 Multi-party collaboration in natural disaster emergency response

Effective natural disaster response requires coordination among multiple stakeholders to integrate diverse resources and ensure timely and efficient action. Existing studies highlight that governments, international organizations, healthcare institutions, and enterprises each play indispensable roles in disaster response, and that their coordinated actions are crucial for achieving successful recovery and resilient outcomes.

#### 2.1.1 Role and responsibilities of government

Governments play a central role in natural disaster emergency response. A study covering 90 countries from 1995 to 2019 (8) found that improving disaster resilience significantly reduces disaster-induced losses. Governments enhance resilience through investments in infrastructure, information and communication technology, institutional capacity, food security, women’s empowerment, human capital, and social cohesion. These measures strengthen post-disaster recovery capacity and improve resource coordination, facilitating timely emergency responses. During emergencies, governments act not only as providers and coordinators of resources but also as designers of incentives and policies that encourage participation across social sectors (9). Policy instruments—such as fiscal subsidies, financial support, and legal frameworks—enable governments to mobilize nonprofit organizations, the private sector, and local communities toward collective disaster management (10). Hence, government action must be multifaceted and tightly integrated with other organizations to effectively address the complex challenges disasters pose.

#### 2.1.2 Support and cooperation from international organizations

International organizations provide critical financial, technical, and material support in disaster emergency response. The World Health Organization (WHO) coordinates global emergency health responses, rapidly assessing health conditions, deploying medical assistance, and supporting disease prevention and health promotion (11). The World Food Programme (WFP) delivers emergency food aid to affected populations, using early warning, proactive planning, and collaboration with other UN agencies to strengthen disaster management (12, 13). The International Federation of Red Cross and Red Crescent Societies (IFRC) plays a vital role in the early stages of disasters by offering emergency relief, advocating for legal frameworks, and protecting vulnerable groups (14, 15). The United Nations Office for the Coordination of Humanitarian Affairs (OCHA) oversees coordination, information management, policy guidance, and resource mobilization (16). The United Nations Children’s Fund (UNICEF) safeguards children’s health and access to water and sanitation (17). Through close collaboration and coordination, these international organizations ensure that humanitarian aid and health interventions are delivered rapidly and equitably.

#### 2.1.3 Emergency response capacity of healthcare institutions

Healthcare institutions are indispensable in disaster management, responsible for emergency medical treatment, triage, infection control, psychological support, logistics, and post-disaster rehabilitation. Their effectiveness directly affects the timeliness and quality of healthcare delivery after disasters. Coordination with government agencies, international organizations, NGOs, and private sectors is vital for maximizing efficiency (18). Nonetheless, healthcare institutions face major challenges such as infrastructure damage, supply shortages, communication breakdowns, and workforce strain (19). Strengthening preparedness and resilience through risk assessment, training, infrastructure upgrades, and inter-agency cooperation is essential (20). Since medical facilities rely on broader urban systems—such as power, water, and transport—institutions should plan redundancies, allocate budgets strategically, and invest in training to bolster resilience (21). Simulation-based training and clear role definitions further enhance emergency response capacity (22). Moreover, coordination mechanisms and shared training between organizations can improve systemic preparedness and operational efficiency (16).

#### 2.1.4 Enterprises’ supply of materials and technical support

Enterprises play a crucial role in modern disaster response through their strengths in supply chain management, logistics, and information technology. They contribute by maintaining emergency reserves, optimizing production and distribution, and improving logistics networks for rapid delivery (23). Efficient communication and data-sharing systems allow real-time monitoring of supply and demand. Firms can also provide storage facilities and managerial expertise to support public and humanitarian efforts, engage in market-based resource allocation, and fulfill corporate social responsibility while maintaining profitability (24). Technologically, enterprises employ GIS, satellite communications, drones, and AI-based analytics to improve situational awareness and decision-making (25–27). Collaboration between enterprises and governments enhances efficiency, reduces costs, and strengthens disaster resilience. Some studies have proposed government–enterprise collaboration models, emphasizing multi-stage planning (procurement, pre-positioning, distribution) to improve system flexibility and resource allocation (28).

### 2.2 Application of evolutionary game theory in multi-party collaboration

Evolutionary game theory (EGT) provides a rigorous framework for analyzing how stakeholders adjust strategies over time in complex, dynamic environments. In disaster response, EGT reveals how parties adapt their behaviors under different incentives and constraints, seeking stable and efficient cooperation.

#### 2.2.1 Overview of evolutionary game theory

GT originated in the 1960s but gained prominence with John Maynard Smith and George Price’s seminal 1973 paper “The Logic of Animal Conflict” in Nature, which introduced the concept of the Evolutionarily Stable Strategy (ESS) (29, 30). They demonstrated that strategy evolution is often non-rational and dynamic, as participants adjust their decisions through repeated interactions. This concept has since been widely applied in biology, ecology, economics, and social sciences. For example, Hardy and Mesterton-Gibbons studied wasp competition (31), Kuhn et al. analyzed microbial interactions (32), and Stein et al. developed Stackelberg evolutionary games for resource management (33). Domingos et al. explored decision-making in human–machine interaction using EGT (34). Collectively, these studies illustrate EGT’s broad utility for understanding strategic adaptation in complex systems.

#### 2.2.2 Application of evolutionary game theory in emergency response

Recent research increasingly applies EGT to emergency management, particularly in multi-party coordination and decision-making. By modeling behavioral strategies and dynamic interactions, EGT helps optimize cooperation and resource allocation under uncertainty. Applications include modeling stakeholder cooperation, designing incentive mechanisms, and analyzing system stability. For example, Fan et al. integrated EGT with system dynamics to examine public health emergency management (35). Wang et al. developed a tripartite model for emergency supply chain coordination during pandemics (36). Wang et al. used EGT to analyze collaboration among enterprises, regulators, and safety assessment agencies (37). Lv et al. applied a delayed SEIR–EGT model to study panic propagation (38). Yuan et al. used behavioral theories to assess decision-making under emergencies (39). Despite these advances, challenges remain—simplified assumptions, limited empirical data, and lack of behavioral integration constrain applicability. Future research should combine empirical evidence, interdisciplinary methods, and adaptive modeling to improve policy relevance and practical utility.

### 2.3 The influence of public policy on multi-party collaboration

Public policy plays a decisive role in shaping the dynamics of multi-party collaboration, particularly in the context of natural disaster emergency response. Well-designed and effectively implemented policies can align stakeholder incentives, mitigate conflicts over resources, and enhance both the efficiency and sustainability of disaster management systems. Since cooperation is often hindered by conflicting interests, asymmetric information, and varying risk perceptions, public policy provides a crucial institutional framework that guides all parties toward coordinated action.

#### 2.3.1 Policy design and implementation

The quality of policy design directly influences the effectiveness of inter-organizational collaboration during disasters. By leveraging diverse policy tools—such as fiscal subsidies, tax incentives, legislative measures, and resource allocation mechanisms—governments can rapidly mobilize stakeholders and minimize friction arising from uneven resource distribution. Financial subsidies, for example, not only provide direct economic support during emergency response but also facilitate post-disaster reconstruction by incentivizing participation from enterprises and healthcare institutions (40). Legal and regulatory frameworks, in turn, establish clear behavioral norms for all participants, strengthening accountability and deterring opportunistic or self-interested actions that might undermine cooperation (41). However, effective policy implementation requires more than formal regulation or financial intervention—it also demands a nuanced understanding of stakeholder incentives, needs, and willingness to collaborate (42). Empirical studies show that successful policy frameworks balance diverse stakeholder interests to ensure that resources are allocated fairly and efficiently. Governments must also account for the needs of vulnerable populations, ensuring equitable access to medical care and essential services during emergencies (43). Additionally, implementation transparency is critical: if policy enforcement lacks openness or oversight, information asymmetry may emerge, weakening inter-agency trust and reducing the willingness to cooperate (44). Therefore, transparent communication, performance disclosure, and participatory monitoring are essential to maintaining effective collaboration.

#### 2.3.2 Incentive mechanisms and constraints

Incentive mechanisms and constraints are fundamental components of public policy that jointly foster sustained multi-party cooperation. Incentives—including monetary rewards, reputational benefits, and institutional recognition—encourage proactive participation and innovation in disaster response, while constraints—such as legal obligations, performance audits, and penalty systems—ensure accountability and compliance with cooperative agreements. Studies have demonstrated that governments can substantially enhance synergy and operational efficiency by designing balanced systems of rewards and sanctions in public health and emergency management (45). For example, additional funding and commendations can be provided to healthcare institutions demonstrating exceptional performance during crises, whereas noncompliant enterprises or agencies may face penalties or reduced support. These mechanisms motivate continuous improvement and help institutionalize responsible behavior.

Beyond financial incentives, non-material motivations—such as social responsibility, professional ethics, and public reputation—are equally powerful (46). Many enterprises and healthcare institutions participate in disaster relief not solely for profit but also to strengthen their social legitimacy and public trust. Governments can amplify these motivations through media recognition, certification programs, or award systems that elevate the public image of participating entities. Meanwhile, regulatory constraints are indispensable for ensuring that cooperation agreements are followed in practice. Laws, contracts, and administrative guidelines can clearly define the rights and responsibilities of each stakeholder, while mechanisms such as public performance reporting or compliance scorecards foster transparency and societal oversight (47). Governments can further issue standard operating procedures (SOPs) to unify emergency actions across regions and establish accountability systems to prevent negligence and moral hazard (9). In essence, well-calibrated incentives and constraints operate synergistically to ensure effective and sustained collaboration. Policymakers must carefully balance these two forces—reward and regulation—to maintain stakeholder engagement, prevent coordination failures, and enhance the long-term resilience of the disaster management system.

Recent empirical studies in Asia further demonstrate how effective cross-sectoral coordination can enhance disaster resilience and public health outcomes. For instance, recent empirical research in Asia further illustrates the value of cross-sectoral coordination in managing compound crises. Kim et al. showed how Singapore leveraged long-standing partnerships among government agencies, enterprises, and healthcare institutions to enhance adaptive governance during COVID-19 (48). Similarly, Dutta and Fischer analyzed rural India’s decentralized disaster governance and found that multi-level collaboration between local authorities, health services, and civil society improved community-level resilience (49). Mitra and Shaw emphasized the need for integrated disaster governance frameworks in Asia, highlighting the importance of synchronized public–private cooperation in managing systemic risks (50). These findings reinforce the practical relevance of the four-party game framework by demonstrating how intersectoral collaboration can enhance response capacity and resilience in real-world disaster contexts.

In summary, public policy functions as the structural backbone of multi-party collaboration in disaster response. Through deliberate policy design, transparent implementation, and balanced incentive–constraint mechanisms, governments can transform fragmented efforts into coordinated, adaptive, and equitable disaster management systems. This institutional perspective also provides the theoretical foundation for the four-party evolutionary game model developed in this study.

## 3 Construction of a four-party evolutionary game model

### 3.1 Description of the game problem

Global natural disaster health emergency response is a complex, multi-layered system involving numerous stakeholders with interdependent interests in resource investment, policy formulation, coordinated action, and operational execution. This study focuses on four key actors—government, international organizations, healthcare institutions, and enterprises—and explores the synergy and conflicts among them in responding to natural disasters. To clarify, the term “government” in our model refers primarily to national-level agencies responsible for disaster management and health emergency coordination. While local and provincial governments play critical operational roles on the ground, national governments are typically in charge of inter-sectoral policy integration, international collaboration, and macro-level resource allocation. The term “enterprises” encompasses private-sector organizations that contribute materially or technologically to disaster response and recovery, including logistics providers, pharmaceutical manufacturers, construction firms, and technology companies. We chose these four institutional actors because they collectively represent the core pillars of organized disaster response at the policy and operational levels. Although stakeholders such as the public, NGOs, or media play meaningful roles, we excluded them from this game-theoretic model to retain tractability and focus on strategic institutional interactions. These exclusions are discussed further in the limitations section. By delving into these intricate cooperation dynamics, the research aims to uncover how stakeholders can, through evolving collaborative mechanisms, jointly confront the challenges posed by disasters and achieve resource sharing and coordinated action.

Governments play a central role in emergency response, encompassing national emergency management agencies, local governments in disaster-stricken areas, health administration departments, the police, and firefighting units. Their responsibilities include formulating policies and regulations, organizing resource allocation, and overseeing post-disaster coordination. The primary governmental objective is to ensure the safety and stability of affected regions, restore public services, and mobilize support from diverse stakeholders. However, given limited resources, governments must make optimal allocation decisions among domestic and international organizations, healthcare institutions, and enterprises. Challenges arise in supervising and incentivizing enterprises and healthcare institutions, as well as addressing disparities in resource distribution and shortcomings in policy execution.

Healthcare institutions form a vital component of post-disaster emergency response, incorporating hospitals of varying levels, emergency medical teams, on-site rescue centers, and professional medical associations. Their core functions involve delivering emergency care, treatment, and long-term rehabilitation services to disaster victims, alongside coordinating with other rescue agencies. Within the bounds of funding and resources provided by the government, healthcare institutions must maximize rescue efficiency to ensure the timeliness and quality of medical services. They also face decisions regarding whether and how to integrate assistance from international organizations, such as funding or technical support, particularly when resources are constrained.

International organizations—such as the World Health Organization, the World Food Programme, the International Red Cross and Red Crescent Movement, NGOs, and other humanitarian agencies—contribute critically in the aftermath of disasters by offering monetary support, technical expertise, and coordinated aid to help governments and healthcare institutions mitigate the impacts of natural disasters. Typically possessing cross-national operational capacities and abundant aid resources, these organizations can promptly deliver assistance. Their decision-making involves selecting aid recipients and types of support, as well as assessing stakeholder needs to ensure efficient and fair distribution of aid resources. Concurrently, international organizations must collaborate closely with governments and healthcare institutions to guarantee effective transfer of aid to disaster zones and uphold principles of equity, while also considering the sustainable use of resources.

Enterprises primarily supply crucial materials and technical solutions in post-disaster scenarios—ranging from pharmaceutical and medical device manufacturers to logistics and transportation providers, as well as construction firms engaged in rebuilding efforts. They assist governments, healthcare institutions, and others in addressing urgent post-disaster demands. Enterprises weigh whether—and how much—to invest in disaster relief resources, balancing cost-effectiveness, social responsibility, and profit objectives when deciding on the quantity and quality of donated materials and services. Furthermore, they must establish effective partnerships with governments and international organizations, particularly in urgent situations where rapid response and appropriate technological support are vital to prevent conflicts arising from resource shortages.

Although the general public plays a vital role in disaster preparedness and response—particularly in shaping community resilience, compliance, and information dissemination—we chose not to include it as a standalone player in this evolutionary game. This modeling decision stems from the complexity of quantifying public behavior in a multi-agent strategic framework, especially when focusing on institutional decision-making and inter-organizational dynamics. Nevertheless, the public’s role is indirectly reflected through the payoffs and strategic incentives of government, healthcare institutions, and enterprises, whose actions are often guided by public expectations, social pressure, and accountability mechanisms. We explicitly acknowledge this limitation in the conclusion and propose future model extensions that incorporate public participation more directly.

**Figure 1:**
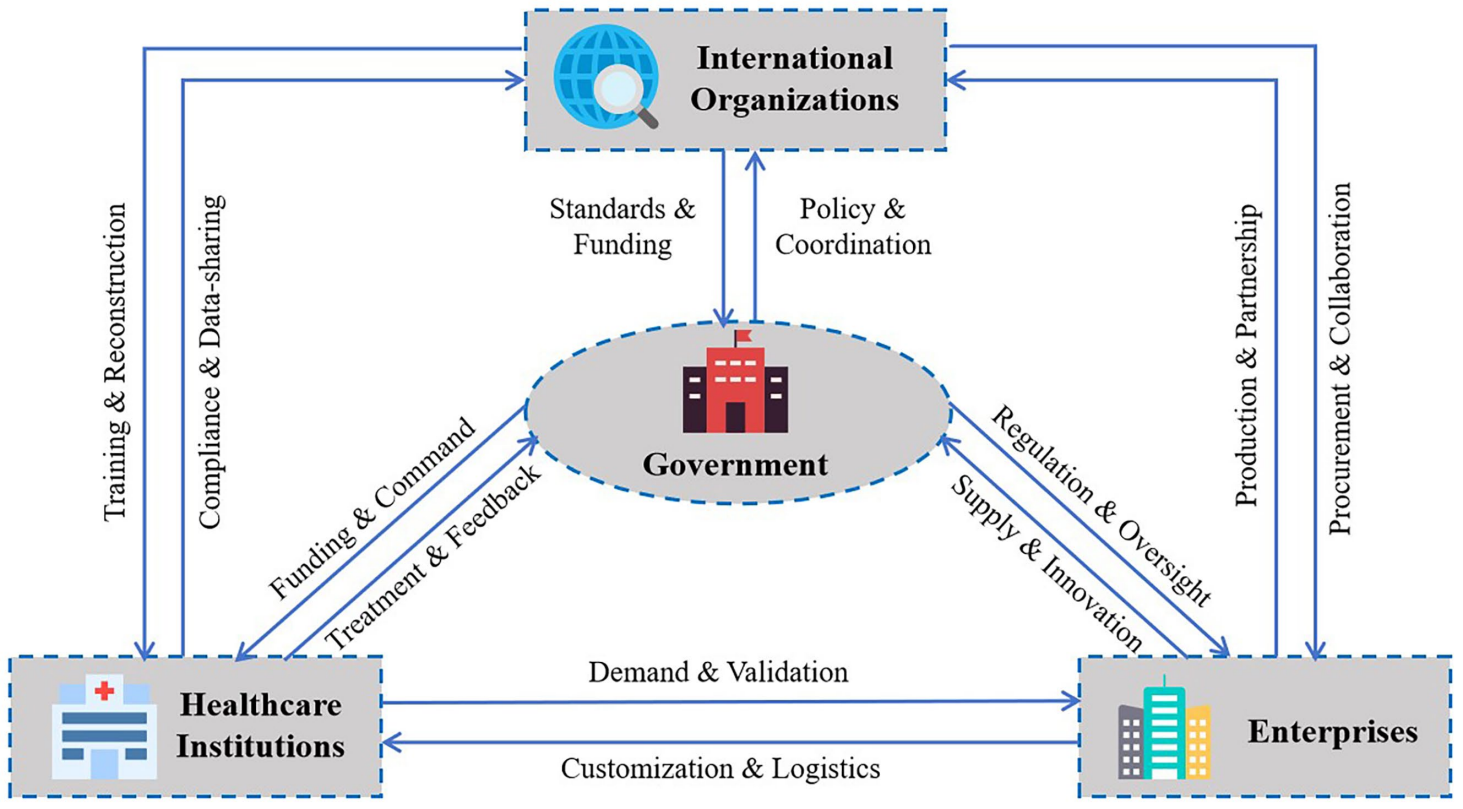
Interaction network of four actors (G = Government, I = International Orgs, H = Healthcare, E = Enterprises).

These game-related issues reflect the multifaceted interactions and collaborative relationships among stakeholders in post-disaster emergency response. As the central actor, government must reconcile multiple interests, ensure rational and efficient distribution of resources, and coordinate the operations of international organizations, healthcare institutions, and enterprises to maximize the overall effectiveness of post-disaster rescue efforts. Figure 1 illustrates the logical relationships among the four key actors in the natural disaster emergency response collaborative evolutionary game.

The institutional relationships and strategic assumptions in this study are informed by the disaster response practices in China, where the government plays a central role in resource mobilization and policy coordination. However, the model structure remains adaptable to other governance contexts, such as the United States, by adjusting payoff parameters and role strengths. For example, in a decentralized federal system, strategic dominance may shift from central government to local authorities or private-sector actors. The assumptions are thus intended as a stylized but flexible abstraction rather than a country-specific model.

### 3.2 Basic assumptions

From a public policy perspective, we construct an evolutionary game model involving the Government (G), International Organizations (I), Healthcare Institutions (H), and Enterprises (E). We make the following assumptions:

- Government Assumption (GA): The government faces two strategy options: “Strengthened Regulation” (G_1_) or “Market Deregulation” (G_2_). Strengthened regulation may yield more efficient resource allocation in the short term, but potentially dampens market vitality. Deregulation could boost market mechanisms, yet may create imbalances in emergency resource distribution. We assume the government’s primary objective is to optimize resource allocation, maximize social welfare, and balance control versus market autonomy when natural disasters occur. Let the probability of the government choosing “Strengthened Regulation” be x ( 0≤x≤1 ) and the probability of choosing “Market Deregulation” be 1−x . This assumption reflects the government’s real-world responsibility to navigate the trade-off between centralized coordination and economic flexibility in times of crisis.
- International Organizations Assumption (IA): International organizations may opt for “Direct Intervention” (I_1_) or “Indirect Coordination” (I_2_). Direct intervention can swiftly provide assistance but may engender dependence; indirect coordination, by contrast, focuses on aiding collaboration between governments and market entities to enhance long-term governance capacity, though it risks slower response. We assume international organizations aim to foster global cooperation and sustainable post-disaster recovery, thereby supporting stakeholders in building resilient emergency response systems. Let the probability of choosing “Direct Intervention” be y (0≤y≤1) and “Indirect Coordination” be 1−y . This assumption captures the dual mandate of international actors to provide immediate relief while promoting long-term resilience in host countries.
- Healthcare Institutions Assumption (HA): Healthcare institutions choose between “Public Welfare First” (H_1_) or “Cost Control” (H_2_). Emphasizing public welfare increases attention to social responsibility and public health but may raise operational costs; focusing on cost control can promote operating efficiency but could undermine the quality and equity of emergency medical services. We assume that healthcare institutions strive to optimize resource usage and cost-effectiveness while safeguarding public health. Let the probability of choosing “Public Welfare First” be z(0≤z≤1) and “Cost Control” be 1−z . This assumption reflects the operational reality that medical institutions must balance financial constraints with ethical obligations during crises.
- Enterprise assumption (EA): Enterprises choose between “Corporate Social Responsibility” (E_1_) or “Profit Maximization” (E_2_). Fulfilling social responsibility may impose additional social costs, whereas pursuing profit maximization could drive short-term economic benefits at the expense of broader social and environmental considerations. We assume enterprises aim to balance social responsibility and profitability, supporting economic recovery while realizing sustainable long-term business interests. Let the probability of choosing “Corporate Social Responsibility” be w (0≤w≤1) and “Profit Maximization” be 1−w . This assumption reflects real-world corporate decision-making under public pressure, regulatory incentives, and market conditions during emergencies. Table 1 lists the main parameters of the evolutionary game model for each stakeholder, including costs and benefits under different strategy choices. These assumptions and parameters form the foundation of the four-party evolutionary game model. By specifying each stakeholder’s strategic choices, costs, and benefits, the model can analyze how government, international organizations, healthcare institutions, and enterprises dynamically interact under different configurations of public policy.

It is important to note that although the assumptions are presented separately for clarity, the decision-making processes of the stakeholders are interdependent in practice and within the game model. Resource allocation is modeled as a dynamic outcome of strategic interactions, where each player’s payoff depends not only on their own actions but also on the strategies of others. For example, a government’s decision to regulate influences enterprise participation; healthcare institutions’ efficiency is shaped by both public funding and enterprise support. While international organizations operate with greater autonomy, their interventions are still influenced by observed needs and institutional responses. Thus, the model implicitly reflects the coupling of resource allocation behaviors through payoff structures and replicator dynamics, even if the assumptions are structured actor by actor.

### 3.3 Construction of the payoff matrix

Based on the assumptions and parameter settings outlined previously, we can derive the evolutionary game payoff matrix for the four players—Government (G) choosing from {Strengthened Regulation (G_1_), Market Deregulation (G_2_)}, International Organizations (I) choosing from {Direct Intervention (I_1_), Indirect Coordination (I_2_)}, Healthcare Institutions (H) choosing from {Public Welfare (H_1_), Cost Control (H_2_)}, and Enterprises (E) choosing from {Social Responsibility (E_1_), Profit Maximization (E_2_)}. Table 2 presents the resulting payoff matrix.

**Table 2:** Evolutionary game payoff matrix for government, international organizations, healthcare institutions, and enterprises.

No.	Strategy combination	Government payoff	International organizations payoff	Healthcare institutions payoff	Enterprises payoff
1	{G_1_, I_1_, H_1_, E_1_}	B_1_-C_1_	B_3_-C_3_ + A	B_5_-C_5_ + U	B_7_-C_7_ + T
2	{G_1_, I_1_, H_1_, E_2_}	B_1_-C_1_ + P	B_3_-C_3_ + A	B_5_-C_5_ + U	B_8_-C_8_
3	{G_1_, I_1_, H_2_, E_1_}	B_1_-C_1_ + P	B_3_-C_3_ + A	B_6_-C_6_	B_7_-C_7_
4	{G_1_, I_1_, H_2_, E_2_}	B_1_-C_1_ + P	B_3_-C_3_ + A	B_6_-C_6_-R_2_	B_8_-C_8_-R_3_
5	{G_1_, I_2_, H_1_, E_1_}	B_1_-C_1_	B_4_-C_4_ + A	B_5_-C_5_ + U	B_7_-C_7_ + T
6	{G_1_, I_2_, H_1_, E_2_}	B_1_-C_1_ + P	B_4_-C_4_ + A	B_5_-C_5_ + U	B_8_-C_8_-R_3_
7	{G_1_, I_2_, H_2_, E_1_}	B_1_-C_1_ + P	B_4_-C_4_ + A	B_6_-C_6_	B_7_-C_7_
8	{G_1_, I_2_, H_2_, E_2_}	B_1_-C_1_ + P	B_4_-C_4_ + A	B_6_-C_6_-R_2_	B_8_-C_8_-R_3_
9	{G_2_, I_1_, H_1_, E_1_}	B_2_-C_2_	B_3_-C_3_-D + A	B_5_-C_5_ + U	B_7_-C_7_
10	{G_2_, I_1_, H_1_, E_2_}	B_2_-C_2_-R_1_-S	B_3_-C_3_-D + A	B_5_-C_5_	B_8_-C_8_
11	{G_2_, I_1_, H_2_, E_1_}	B_2_-C_2_-R_1_-S	B_3_-C_3_-D	B_6_-C_6_-R_2_	B_7_-C_7_
12	{G_2_, I_1_, H_2_, E_2_}	B_2_-C_2_-R_1_-S	B_3_-C_3_-D	B_6_-C_6_-R_2_	B_8_-C_8_-R_3_
13	{G_2_, I_2_, H_1_, E_1_}	B_2_-C_2_	B_4_-C_4_ + A	B_5_-C_5_ + U	B_7_-C_7_
14	{G_2_, I_2_, H_1_, E_2_}	B_2_-C_2_-R_1_-S	B_4_-C_4_ + A	B_5_-C_5_	B_8_-C_8_
15	{G_2_, I_2_, H_2_, E_1_}	B_2_-C_2_-R_1_-S	B_4_-C_4_	B_6_-C_6_-R_2_	B_7_-C_7_
16	{G_2_, I_2_, H_2_, E_2_}	B_2_-C_2_-R_1_-S	B_4_-C_4_	B_6_-C_6_-R_2_	B_8_-C_8_-R_3_

Each row corresponds to a particular combination of strategies chosen by the four players. The associated payoff formulas on the right columns indicate the net returns each player obtains given that specific strategy profile. These payoff expressions incorporate both the benefits and costs determined by the parameters introduced earlier, thereby reflecting the interplay of incentives and penalties under different policy settings.

## 4 Stability analysis of strategies in the four-party game

**Table 1:** Parameters of the evolutionary game model for government (G), international organizations (I), healthcare institutions (H), and enterprises (E).

Actor	Parameter	Meaning	Influencing Factors	Influence mechanism
Government (G)	$B_{1}$	Benefit from strengthened regulation	Government strengthens regulation ( x )	More efficient short-term resource allocation and a stable public health & medical relief system
	C_1_	Cost of strengthened regulation	Government strengthens regulation ( x )	Increased regulatory costs, including administrative expenses, manpower, and implementation mechanisms
	B_2_	Benefit from market deregulation	Government deregulates ( 1−x )	Increased market vitality, allowing enterprises to optimize resource allocation autonomously and stimulating economic growth
	C_2_	Cost of market deregulation	Government deregulates ( 1−x )	Potential imbalance in resource allocation and regulatory gaps, affecting post-disaster recovery capacity
	R_1_	Losses from regulatory failure	Government deregulates ( 1−x ), enterprise pursues profit maximization ( 1−w ), healthcare institutions prioritize cost control ( 1−z )	Insufficient regulation leads to imbalanced distribution of emergency resources, inadequate medical services, and opportunistic pursuit of profit by enterprises
	S	Cost of strengthening regulation post-failure	Government deregulates ( 1−x ), enterprise and healthcare institutions reduce social responsibility ( 1−w , 1−z )	Additional resources required to readjust market mechanisms after an initial failure in regulation
	P	Level of regulatory penalties	Government strengthens regulation ( x ), enterprises reduce social responsibility ( 1−w ), healthcare institutions reduce public welfare ( 1−z )	More stringent regulation raises compliance costs for enterprises and healthcare institutions
International Organizations (I)	B_3_	Benefit of direct intervention	Organization chooses direct intervention ( y )	Rapid provision of emergency assistance to mitigate disaster impact
	C_3_	Cost of direct intervention	Organization chooses direct intervention ( y )	High financial outlays for humanitarian aid and logistical coordination
	B_4_	Benefit of indirect coordination	Organization chooses indirect coordination ( 1−y )	Long-term capacity-building and policy collaboration, improving governance structures over time
	C_4_	Cost of indirect coordination	Organization chooses indirect coordination ( 1−y )	Requires time and resources to develop policies and coordinate among different countries and institutions
	D	Dependency losses from excessive intervention	Organization chooses direct intervention ( y ), government regulation is weak ( 1−x )	May increase reliance on international assistance, reducing the autonomy of local government and market actors
	A	Willingness of international organizations to provide aid	Government regulatory intensity ( x ), healthcare institutions’ commitment to public welfare ( z )	When government regulation is robust and healthcare service quality is high, international organizations are more inclined to offer assistance
Healthcare Institutions (H)	B_5_	Benefit from public welfare strategy	Institutions choose public welfare ( z )	Enhanced service quality, eligibility for government subsidies, and higher social recognition
	C_5_	Cost of public welfare strategy	Institutions choose public welfare ( z )	Increased operational expenses, including free medical services and additional manpower/materials
	B_6_	Benefit of cost control strategy	Institutions choose cost control ( 1−z )	Improved operational efficiency, cost savings, and better financial performance
	C_6_	Cost of cost control strategy	Institutions choose cost control ( 1−z )	Potential decline in medical service quality and reduced patient satisfaction
	R_2_	Public health loss	Government deregulates ( 1−x ), healthcare institutions choose cost control ( 1−z ), enterprises pursue profit maximization ( 1−w )	Leads to inadequate medical resources and compromised public health outcomes
	U	Efficiency of resource utilization in healthcare institutions	Government regulatory intensity ( x ), enterprise social responsibility ( w )	Strong government regulation and socially responsible enterprises improve resource allocation efficiency in healthcare institutions
Enterprises (E)	B_7_	Benefit of fulfilling social responsibility	Enterprises choose social responsibility ( w )	Better brand reputation, eligibility for government subsidies, and broader social support
	C_7_	Cost of fulfilling social responsibility	Enterprises choose social responsibility ( w )	Additional funding for public welfare initiatives, donations, etc.
	B_8_	Benefit of profit maximization	Enterprises choose profit maximization ( 1−w )	Higher short-term economic returns and greater shareholder value
	C_8_	Cost of profit maximization	Enterprises choose profit maximization ( 1−w )	Possible exposure to lawsuits and stricter market regulations
	R_3_	Compliance cost due to corporate misconduct	Government strengthens regulation ( x ), enterprises prioritize profit ( 1−w ), healthcare institutions reduce public welfare ( 1−z )	Leads to high compliance costs and risk of market sanctions for enterprises
	T	Market trust in enterprises	Government regulatory intensity ( x ), healthcare institutions’ quality service ( z )	Reasonable regulation and strong healthcare services enhance consumer trust in enterprises

The replicator dynamic equation is a central tool in evolutionary game theory. Rather than predicting the behavior of a single actor, it describes how the proportion of actors adopting a particular strategy changes over time in response to relative payoffs. In the context of disaster emergency response, this means that if one strategy (e.g., strengthened regulation, corporate social responsibility) consistently yields higher benefits than alternatives, its share in the population of decision-makers will grow until an equilibrium is reached. In this way, the replicator dynamic provides a conceptual bridge between micro-level decision rules and macro-level collective outcomes, making it particularly useful for understanding how public policy strategies stabilize—or fail to stabilize—during crises. Building on this framework, we analyze how stakeholders adjust their strategies over time in response to natural disasters. Specifically, we construct replicator dynamic equations for each player—government, international organizations, healthcare institutions, and enterprises—to examine how their choices evolve under different payoff configurations. The detailed derivations of expected payoffs, replicator dynamics, equilibrium conditions, and stability analysis are provided in the Supplementary material.

## 5 Simulation analysis

In the four-party evolutionary game, changes in each parameter can influence the strategic choices made by the players, thereby driving the dynamic evolution of the game and shaping its final outcomes. To validate the correctness and robustness of the four-party game model and to explore how variations in key parameters affect the evolution of outcomes, this study employs Hatlab for numerical simulations. By simulating changes in different parameters, we analyze the trends in and fluctuations of the evolutionary game among the four players (see Table 3).

**Table 3:** Stability analysis of the 16 pure-strategy equilibrium points.

Equilibrium points	λ1	λ2	λ3	λ4	Stability conditions
E_1_(0,0,0,0)	B_5_-B_6_-C_5_ + C_6_ + R_2_	B_7_-B_8_-C_7_ + C_8_ + R_3_	B_3_-B_4_-C_3_ + C_4_-D	B_1_-B_2_-C_1_ + C_2_ + P + R_1_ + S	B_5_-C_5_ < B_6_-C_6_-R_2_, B_7_-C_7_ < B_8_-C_8_-R_3_, B_3_-C_3_-D < B_4_-C_4_, B_1_-C_1_ + P < B_2_-C_2_-R_1_-S
E_2_(1,0,0,0)	B_3_-B_4_-C_3_ + C_4_	B_7_-B_8_-C_7_ + C_8_ + R_3_	B_5_-B_6_-C_5_ + C_6_ + R_2_ + U	B_2_-B_1_ + C_1_-C_2_-P-R_1_-S	B_3_-C_3_ < B_4_-C_4_, B_7_-C_7_ < B_8_-C_8_-R_3_, B_5_-C_5_ + U < B_6_-C_6_-R_2_, B_2_-C_2_-R_1_ < B_1_-C_1_ + P-S
E_3_(0,1,0,0)	B_4_-B_3_ + C_3_-C_4_ + D	B_5_-B_6_-C_5_ + C_6_ + R_2_	B_7_-B_8_-C_7_ + C_8_ + R_3_	B_1_-B_2_-C_1_ + C_2_ + P + R_1_ + S	B_4_-C_4_ < B_3_-C_3_-D, B_5_-C_5_ < B_6_-C_6_-R_2_, B_7_-C_7_ < B_8_-C_8_-R_3_, B_1_-C_1_ + P < B_2_-C_2_-R_1_-S
E_4_(0,0,1,0)	B_7_-B_8_-C_7_ + C_8_	B_3_-B_4_-C_3_ + C_4_-D	B_6_-B_5_ + C_5_-C_6_-R_2_	B_1_-B_2_-C_1_ + C_2_ + P + R_1_ + S	B_7_-C_7_ < B_8_-C_8_, B_3_-C_3_-D < B_4_-C_4_, B_6_-C_6_-R_2_ < B_5_-C_5_, B_1_-C_1_ + P < B_2_-C_2_-R_1_-S
E_5_(0,0,0,1)	B_3_-B_4_-C_3_ + C_4_-D	B_8_-B_7_ + C_7_-C_8_-R_3_	B_5_-B_6_-C_5_ + C_6_ + R_2_ + U	B_1_-B_2_-C_1_ + C_2_ + P + R_1_ + S	B_3_-C_3_-D < B_4_-C_4_, B_8_-C_8_-R_3_ < B_7_-C_7_, B_5_-C_5_ + U < B_6_-C_6_-R_2_, B_1_-C_1_ + P < B_2_-C_2_-R_1_-S
E_6_(1,1,0,0)	B_4_-B_3_ + C_3_-C_4_	B_7_-B_8_-C_7_ + C_8_ + R_3_	B_5_-B_6_-C_5_ + C_6_ + R_2_ + U	B_2_-B_1_ + C_1_-C_2_-P-R_1_-S	B_4_-C_4_ < B_3_-C_3_, B_7_-C_7_ < B_8_-C_8_-R_3_, B_5_-C_5_ + U < B_6_-C_6_-R_2_, B_2_-C_2_-R_1_ < B_1_-C_1_ + P-S
E_7_(1,0,1,0)	B_3_-B_4_-C_3_ + C_4_	B_6_-B_5_ + C_5_-C_6_-R_2_-U	B_7_-B_8_-C_7_ + C_8_ + R_3_ + T	B_2_-B_1_ + C_1_-C_2_-P-R_1_-S	B_3_-C_3_ < B_4_-C_4_, B_6_-C_6_-R_2_ < B_5_-C_5_ + U, B_7_-C_7_ + T < B_8_-C_8_-R_3_, B_2_-C_2_-R_1_ < B_1_-C_1_ + P-S
E_8_(0,1,1,0)	B_7_-B_8_-C_7_ + C_8_	B_4_-B_3_ + C_3_-C_4_ + D	B_6_-B_5_ + C_5_-C_6_-R_2_	B_1_-B_2_-C_1_ + C_2_ + P + R_1_ + S	B_7_-C_7_ < B_8_-C_8_, B_4_-C_4_ < B_3_-C_3_-D, B_6_-C_6_-R_2_ < B_5_-C_5_, B_1_-C_1_ + P < B_2_-C_2_-R_1_-S
E_9_(1,0,0,1)	B_3_-B_4_-C_3_ + C_4_	B_5_-B_6_-C_5_ + C_6_ + U	B_8_-B_7_ + C_7_-C_8_-R_3_	B_2_-B_1_ + C_1_-C_2_-P-R_1_-S	B_3_-C_3_ < B_4_-C_4_, B_5_-C_5_ + U < B_6_-C_6_, B_8_-C_8_-R_3_ < B_7_-C_7_, B_2_-C_2_-R_1_ < B_1_-C_1_ + P-S
E_10_(0,1,0,1)	B_4_-B_3_ + C_3_-C_4_ + D	B_8_-B_7_ + C_7_-C_8_-R_3_	B_5_-B_6_-C_5_ + C_6_ + R_2_ + U	B_1_-B_2_-C_1_ + C_2_ + P + R_1_ + S	B_4_-C_4_ < B_3_-C_3_-D, B_8_-C_8_-R_3_ < B_7_-C_7_, B_5_-C_5_ + U < B_6_-C_6_-R_2_, B_1_-C_1_ + P < B_2_-C_2_-R_1_-S
E_11_(0,0,1,1)	B_1_-B_2_-C_1_ + C_2_	B_8_-B_7_ + C_7_-C_8_	B_3_-B_4_-C_3_ + C_4_-D	B_6_-B_5_ + C_5_-C_6_-R_2_-U	B_1_-C_1_ < B_2_-C_2_, B_8_-C_8_ < B_7_-C_7_, B_3_-C_3_-D < B_4_-C_4_, B_6_-C_6_-R_2_ < B_5_-C_5_ + U
E_12_(1,1,1,0)	B_4_-B_3_ + C_3_-C_4_	B_7_-B_8_-C_7_ + C_8_ + T	B_6_-B_5_ + C_5_-C_6_-R_2_-U	B_2_-B_1_ + C_1_-C_2_-P-R_1_-S	B_4_-C_4_ < B_3_-C_3_, B_7_-C_7_ + T < B_8_-C_8_, B_6_-C_6_-R_2_ < B_5_-C_5_ + U, B_2_-C_2_-R_1_ < B_1_-C_1_ + P-S
E_13_(1,1,0,1)	B_4_-B_3_ + C_3_-C_4_	B_5_-B_6_-C_5_ + C_6_ + U	B_8_-B_7_ + C_7_-C_8_-R_3_	B_2_-B_1_ + C_1_-C_2_-P-R_1_-S	B_4_-C_4_ < B_3_-C_3_, B_5_-C_5_ + U < B_6_-C_6_, B_8_-C_8_-R_3_ < B_7_-C_7_, B_2_-C_2_-R_1_ < B_1_-C_1_ + P-S
E_14_(1,0,1,1)	B_2_-B_1_ + C_1_-C_2_	B_3_-B_4_-C_3_ + C_4_	B_6_-B_5_ + C_5_-C_6_-U	B_8_-B_7_ + C_7_-C_8_-R_3_-T	B_2_-C_2_ < B_1_-C_1_, B_3_-C_3_ < B_4_-C_4_, B_6_-C_6_ < B_5_-C_5_ + U, B_8_-C_8_-R_3_ < B_7_-C_7_ + T
E_15_(0,1,1,1)	B_1_-B_2_-C_1_ + C_2_	B_8_-B_7_ + C_7_-C_8_	B_4_-B_3_ + C_3_-C_4_ + D	B_6_-B_5_ + C_5_-C_6_-R_2_-U	B_1_-C_1_ < B_2_-C_2_, B_8_-C_8_ < B_7_-C_7_, B_4_-C_4_ < B_3_-C_3_-D, B_6_-C_6_-R_2_ < B_5_-C_5_ + U
E_16_ (1)	B_2_-B_1_ + C_1_-C_2_	B_4_-B_3_ + C_3_-C_4_	B_8_-B_7_ + C_7_-C_8_-T	B_6_-B_5_ + C_5_-C_6_-U	B_2_-C_2_ < B_1_-C_1_, B_4_-C_4_ < B_3_-C_3_, B_8_-C_8_-R_3_ < B_7_-C_7_ + T, B_6_-C_6_ < B_5_-C_5_ + U

### 5.1 Evolutionary simulation analysis across different response phases

Drawing on the four-phase framework of strategy evolution for natural disaster health emergency response described earlier (i.e., Stable Preparedness, Immediate Response, Adaptive Adjustment, and Recovery and Reconstruction), we assign distinct parameter values to match each phase. These values, derived from relevant literature and ensuring they satisfy the stable equilibrium conditions for the ESS points in each phase, are shown in Table 4.

**Table 4:** Initial parameter settings for each phase.

Parameter	Stable preparedness (E2)	Immediate response (E6)	Adaptive adjustment (E12)	Recovery and reconstruction (E16)
B_1_	60	90	85	80
C_1_	35	40	40	40
B_2_	40	60	60	60
C_2_	30	30	35	35
R_1_	15	15	15	25
S	8	10	10	15
P	20	40	25	35
B_3_	40	90	75	80
C_3_	25	35	35	40
B_4_	60	60	55	50
C_4_	20	30	30	35
D	20	20	15	25
A	20	40	35	35
B_5_	30	50	80	80
C_5_	25	30	45	50
B_6_	50	90	70	60
C_6_	15	30	35	40
R_2_	10	15	15	30
U	10	10	15	30
B_7_	40	70	70	80
C_7_	25	40	40	50
B_8_	60	90	85	70
C_8_	20	35	35	40
R_3_	10	25	20	30
T	5	30	10	25

Based on these parameter settings, we conduct in-depth simulation analyses in each phase by focusing on the most pertinent three-way interactions at a given time.

#### 5.1.1 Stable preparedness phase

**Figure 2:**
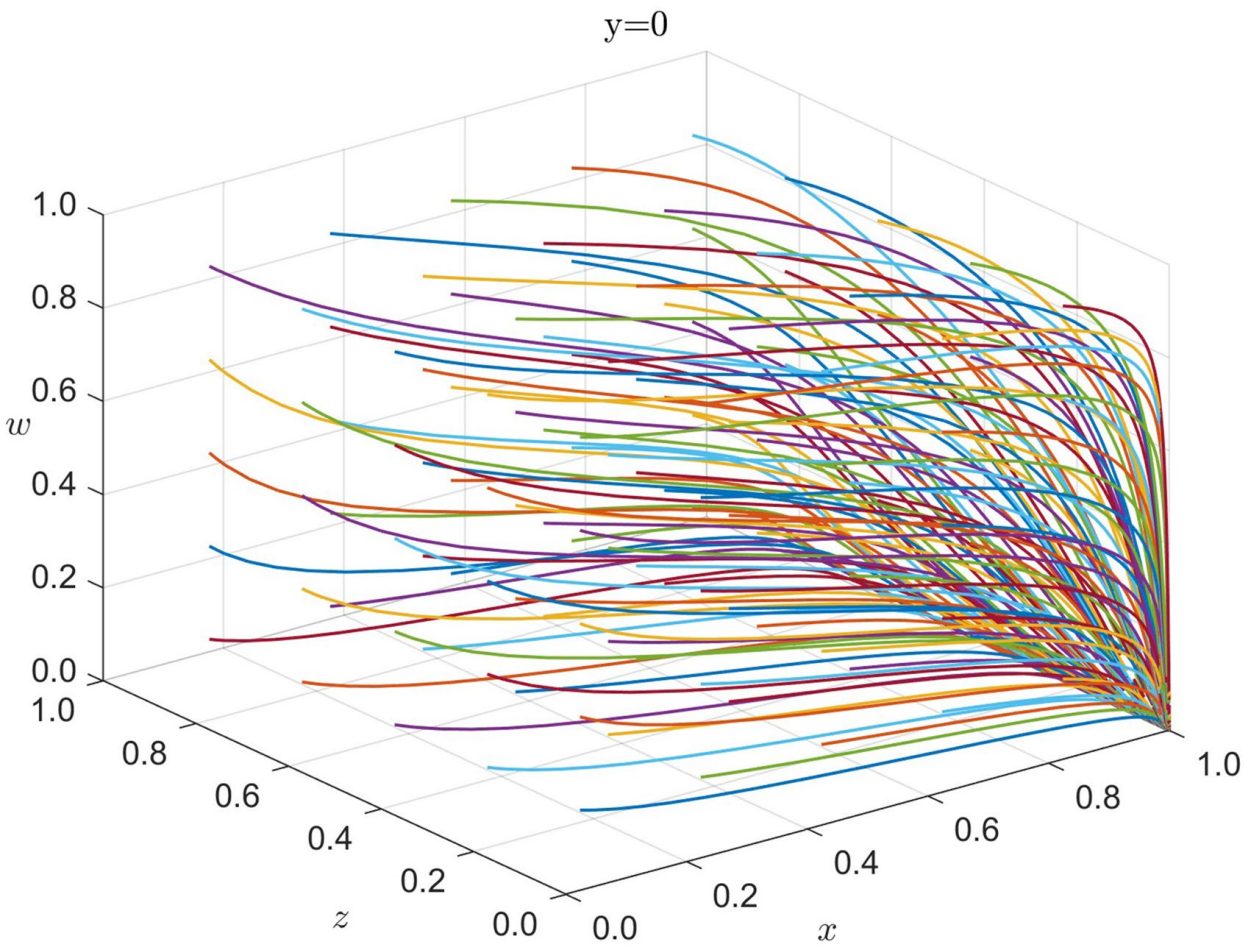
Strategy evolution in stable preparedness (y = 0): x = G, z = H, w = E. Colored lines = different initial conditions.

Before a disaster occurs, the central interactions typically involve the government, healthcare institutions, and enterprises regarding market mechanisms, resource allocation, and healthcare service efficiency. International organizations generally do not intervene proactively in this stage. We therefore simulate the evolutionary game among the government, healthcare institutions, and enterprises. The system eventually stabilizes at $E_{2}(1,0,0,0)$ (as indicated in the figure and text). Figure 2 shows that, during the Stable Preparedness Phase, the government opts for strengthened regulation, while healthcare institutions and enterprises tend toward strategies driven largely by economic interests.

#### 5.1.2 Immediate response phase

**Figure 3:**
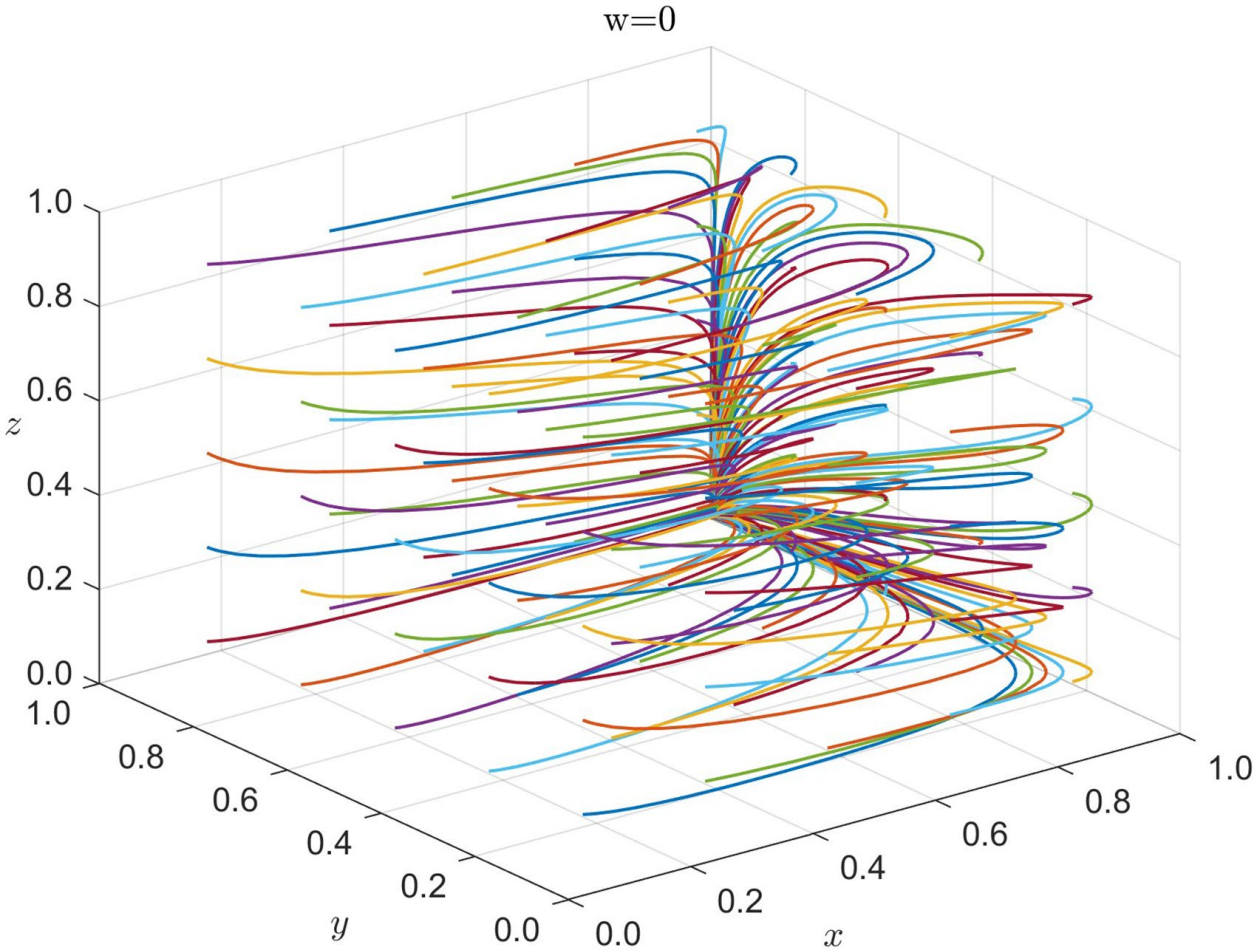
Strategy evolution in immediate response (w = 0): x = G, y = I, z = H. Colored lines = different initial conditions.

Once a disaster strikes, the government responds rapidly, and international organizations swiftly intervene with direct assistance. Healthcare institutions promptly prioritize emergency medical tasks, while the enterprises’ strategies tend to be more singular—focusing on maintaining operational stability with limited strategic adjustment. We therefore simulate the evolutionary game among the government, international organizations, and healthcare institutions. The system eventually stabilizes at $E_{6}(1,1,0,0)$ . Figure 3 illustrates that during the Immediate Response Phase, robust intervention from both the government and international organizations is necessary, and healthcare institutions lean toward cost-control strategies to address shortages in medical resources and the pressure of emergency operations.

#### 5.1.3 Adaptive adjustment phase

**Figure 4:**
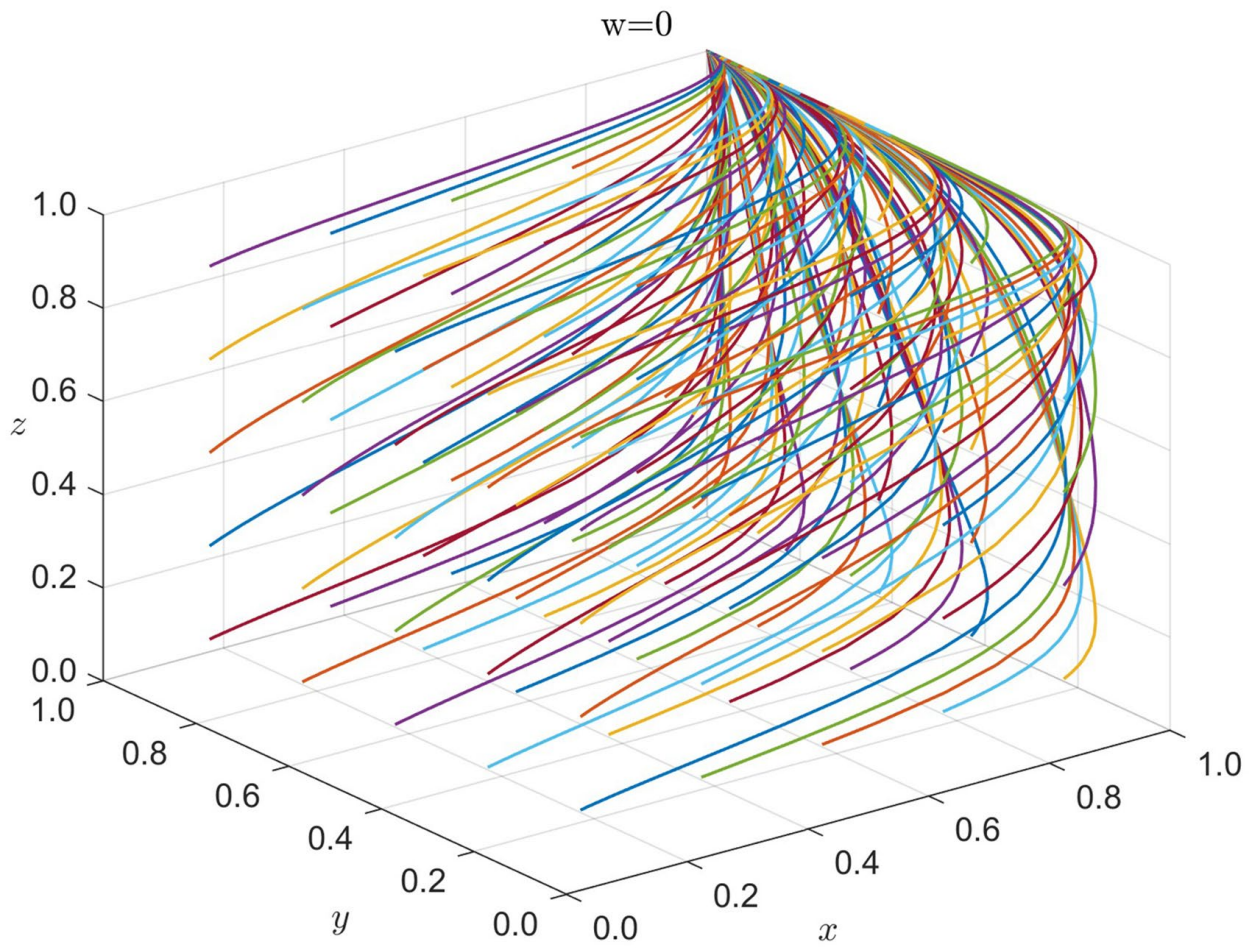
Strategy evolution in adaptive adjustment (w = 0): x = G, y = I, z = H. Colored lines = different initial conditions.

As disaster response proceeds, direct intervention by international organizations becomes relatively stable, and the principal interactions increasingly shift toward the strategies and mutual influence among the government, healthcare institutions, and enterprises—especially regarding the interplay between enterprises’ short-term economic goals and the public welfare orientation of healthcare institutions. We thus simulate the evolutionary game among the government, healthcare institutions, and enterprises. The system ultimately stabilizes at $E_{12}(1,1,1,0)$ , as shown in Figure 4.

#### 5.1.4 Recovery and reconstruction phase

**Figure 5:**
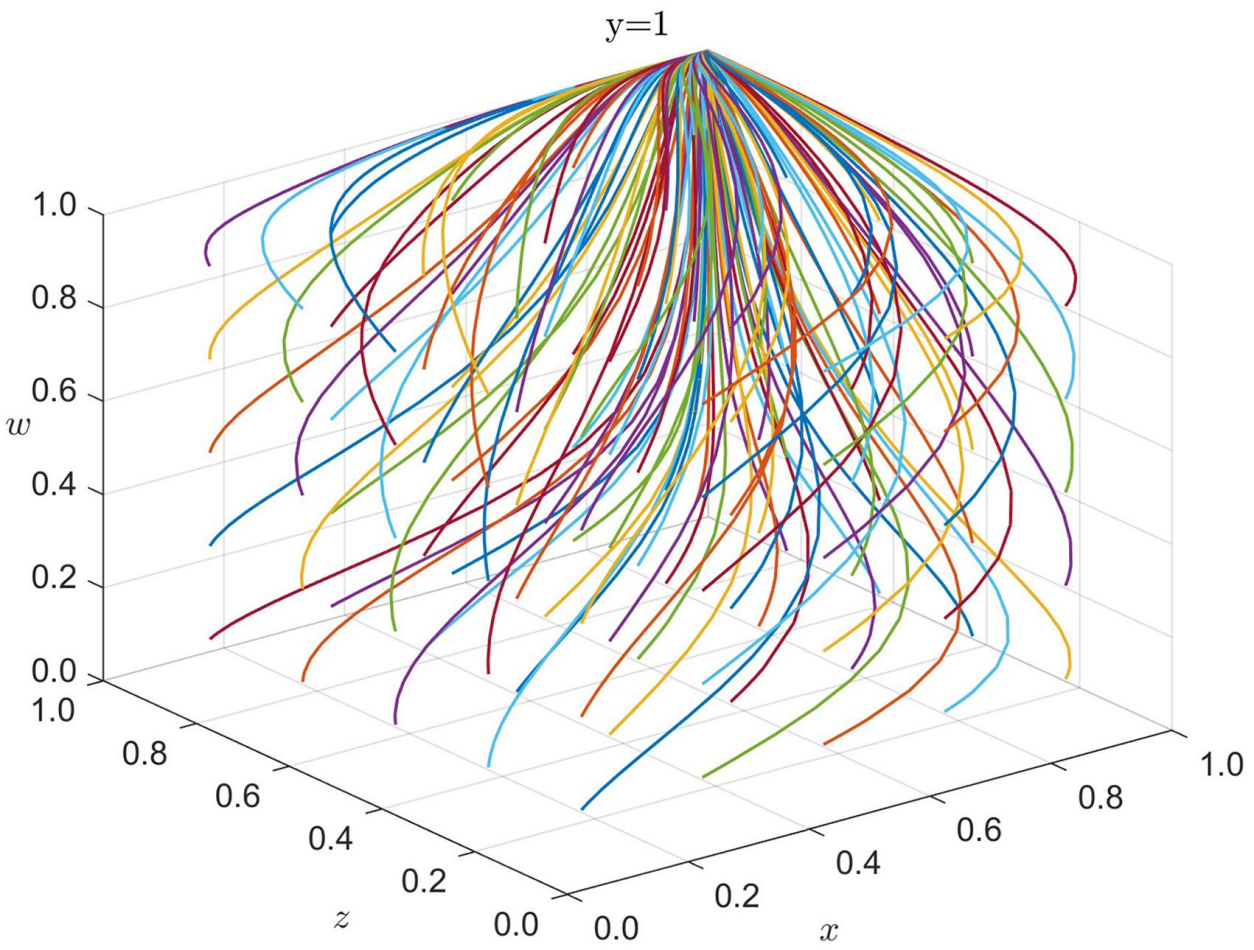
Strategy evolution in recovery phase (y = 1): x = G, z = H, w = E. Colored lines = different initial conditions.

During long-term recovery and reconstruction, the primary drivers of collaboration shift to the government’s policy guidance, healthcare institutions’ public services, and enterprises’ assumption of social responsibility. At this stage, international organizations gradually reduce their level of intervention, and sustained cooperation efforts focus on the government, healthcare institutions, and enterprises. We simulate the evolutionary game among these three stakeholders. The system ultimately settles at $E_{16}(1,1,1,1)$ , as shown in Figure 5.

### 5.2 Sensitivity analysis of key parameters

Building on the ideal-state conditions needed for the system to remain at the equilibrium point $E_{16}$ , and combining insights from the literature with practical considerations, this study selects five critical parameters for sensitivity analysis:

- Government regulatory penalty (P)
- Dependency losses arising from over-intervention by international organizations (D)
- Resource utilization efficiency in healthcare institutions (U)
- Enterprises’ cost of noncompliance $\;(R_{3})$
- Market trust benefits for enterprises (T)

These parameters help clarify the core triggers behind each stakeholder’s strategic choices, shedding light on how changes in policy drive strategic evolution. The goal is to provide more targeted theoretical and practical guidance for policies related to natural disaster emergency response. Initial parameter settings are detailed below.

#### 5.2.1 Sensitivity analysis of government regulatory penalty (P)

**Figure 6:**
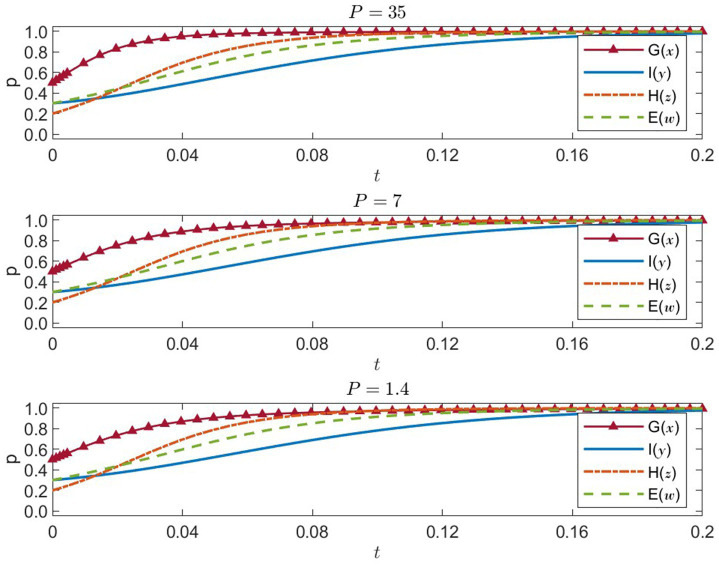
Four-party strategies under government penalty *P* = 35, 7, 1.4 (x = G, y = I, z = H, w = E).

P represents the government’s regulatory penalty, and is set to {35,7,1.4} . The four-party evolutionary trends and outcomes under each value are shown in Figure 6.

1.
P=35 : high regulatory penalty

With a higher penalty in place, the probability that the government chooses “Strengthened Regulation” quickly approaches 1, indicating a substantial rise in the government’s willingness to tighten control. International organizations also converge toward direct intervention, while healthcare institutions and enterprises—driven by stricter regulation—rapidly adopt public-welfare-oriented and socially responsible strategies. Overall, a high penalty effectively incentivizes more responsible actions among all stakeholders.

1.
P=7 : medium regulatory penalty

Although the probability of government regulation remains high, its speed of convergence and eventual equilibrium value are slightly lower than in the *p* = 35 scenario. International organizations exhibit a similar trend, but healthcare institutions and enterprises respond more slowly, suggesting that a medium-level penalty weakens the impetus for these actors to pursue more cooperative or altruistic strategies.

1.
P=1.4 : low regulatory penalty

The government still shows a relatively strong preference for regulation, but healthcare institutions and enterprises move more gradually toward public-welfare and social-responsibility strategies, and the final probabilities for these strategies are lower. This implies that a weak regulatory penalty fails to sufficiently motivate increased social responsibility in healthcare institutions or enterprises. International organizations’ inclination to intervene remains mostly unaffected, but overall cooperation efficiency diminishes.

In summary, higher values of P strengthen governmental oversight and substantially boost healthcare institutions’ and enterprises’ willingness to act in the public interest. Therefore, setting penalty levels appropriately—alongside matching incentive and constraint mechanisms—can effectively enhance fair and efficient allocation of medical resources in natural disaster contexts.

#### 5.2.2 Sensitivity analysis of international organizations’ dependency loss (D)

**Figure 7:**
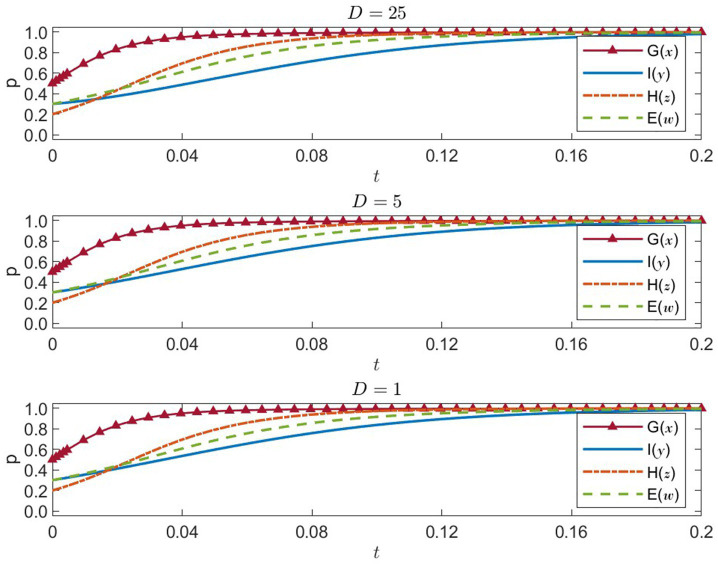
Four-party strategies under international dependency loss D = 25, 5, 1 (x = G, y = I, z = H, w = E).

D stands for the dependency loss caused by excessive intervention from international organizations. We test D={25,5,1} . The simulation results for four-party strategic evolution under these values are presented in Figure 7.

1.
D=25 : high dependency loss

The probability of international organizations choosing direct intervention increases slowly, indicating that heavy dependency costs markedly discourage direct involvement. Although local governments, healthcare institutions, and enterprises still incrementally adopt more positive strategies, the overall convergence rate is slower due to the limited extent of international organizational participation.

1.
D=5 : medium dependency loss

International organizations’ probability of opting for direct intervention rises more quickly, demonstrating a more active stance. Consequently, the strategies of local governments, healthcare institutions, and enterprises also converge faster to stable, positive outcomes. This suggests that moderate dependency losses strike a productive balance, motivating international organizations to intervene and advancing overall strategy evolution.

1.
D=1 : low dependency loss

Here, international organizations’ probability of direct intervention rapidly approaches 1, showing strong willingness for immediate engagement. This highly efficient intervention, in turn, significantly encourages the government to strengthen regulation and prompts healthcare institutions and enterprises to adopt public-welfare- and social-responsibility-oriented strategies, enabling rapid convergence to an ideal equilibrium.

In sum, lowering international organizations’ dependency loss (D) increases their incentive to intervene directly, thereby moving all stakeholders’ strategies more quickly toward the ideal state. Thus, in disaster management, minimizing the negative impact of international organizations’ direct intervention can create a more efficient and coordinated governance framework, optimizing resource allocation and utilization.

#### 5.2.3 Sensitivity analysis of healthcare institutions’ resource efficiency (U)

**Figure 8:**
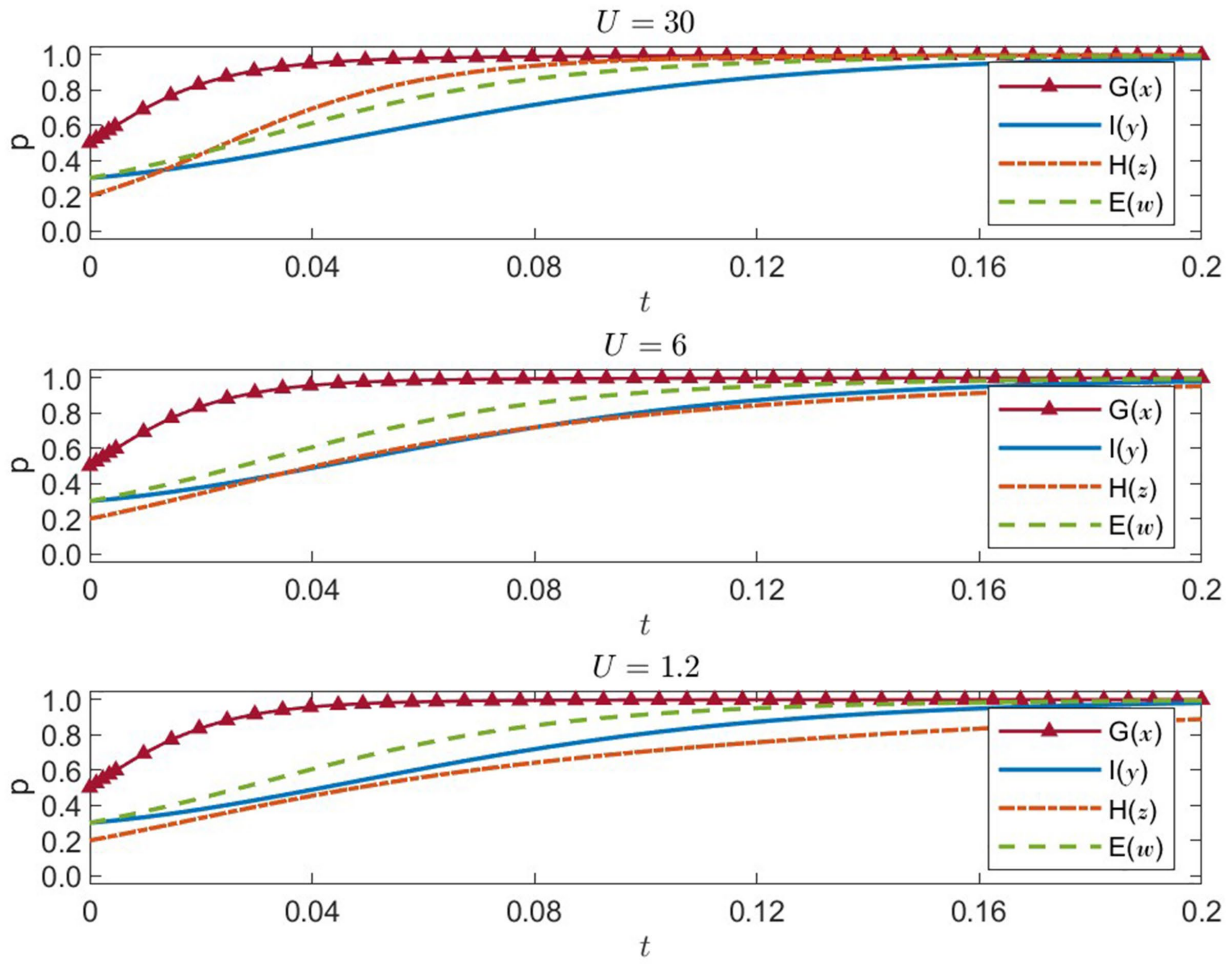
Four-party strategies under healthcare efficiency U = 30, 6, 1.2 (x = G, y = I, z = H, w = E).

U measures the resource utilization efficiency of healthcare institutions. We test U={30,6,1.2} . Simulation outcomes are shown in Figure 8.

1.
U=30 : high resource efficiency

Under high efficiency, the probability that healthcare institutions choose public-welfare-oriented strategies quickly approaches 1, indicating strong motivation to prioritize public health. As healthcare institutions operate effectively, enterprises are likewise spurred to take on greater social responsibility, and both government and international organizations sustain high strategy probabilities. The system converges rapidly to its ideal equilibrium.

1.
U=6 : medium resource efficiency

Healthcare institutions’ probability of choosing public-welfare-first increases at a slower pace, indicating a decline in their impetus for social service. Enterprises also slow in adopting socially responsible strategies. Although government and international organizations remain relatively proactive, the overall time to reach stable equilibrium is slightly extended.

1.
U=1.2 : low resource efficiency

Healthcare institutions’ inclination toward public-welfare strategies noticeably weakens, and enterprises’ willingness to assume social responsibility likewise decreases. Even if government and international organizations maintain positive stances, the shortcomings in healthcare resource utilization significantly hamper the entire system’s convergence to an ideal equilibrium, reducing overall efficiency in medical resource distribution.

Hence, raising healthcare institutions’ resource efficiency (U) stimulates both their own public-welfare motivations and the positive strategy choices of other players. Improving healthcare resource efficiency proves instrumental in enhancing the quality of medical services and resource allocation during natural disaster emergencies.

#### 5.2.4 Sensitivity analysis of enterprises’ cost of noncompliance $\;(R_{3})$

**Figure 9:**
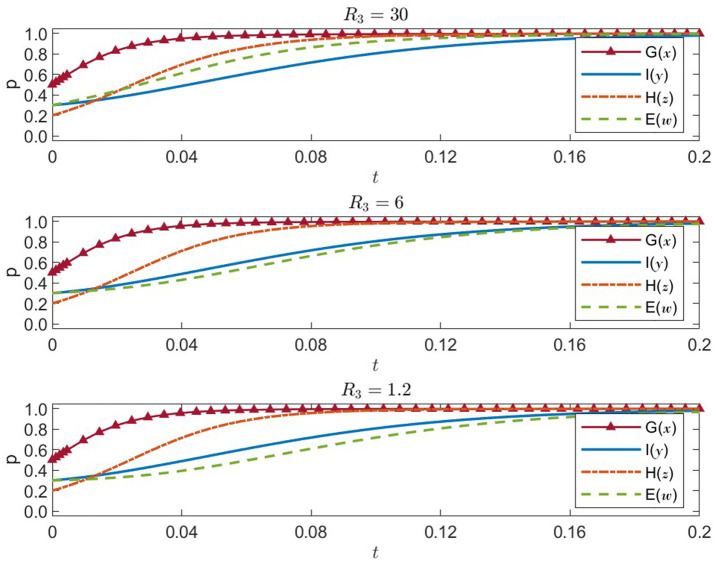
Four-party strategies under enterprise noncompliance cost R₃ = 30, 6, 1.2 (x = G, y = I, z = H, w = E).

$\left(R_{3}\right)$ represents the cost that enterprises incur when violating regulations. We test $\;R_{3}=\{30,6,1.2\}$ . The outcomes appear in Figure 9.

1.
$R_{3}=30$ : high cost of noncompliance

Enterprises quickly adopt socially responsible strategies, with the probability approaching 1, indicating that high noncompliance penalties greatly discourage profit-driven misconduct. This motivates healthcare institutions to embrace public welfare, while government and international organizations also maintain high levels of proactive strategies. The entire system converges swiftly to a desirable equilibrium.

1.
$R_{3}=6$ : medium cost of noncompliance

Enterprises’ social responsibility probability rises more gradually, extending the time needed to stabilize. Their lower motivation to comply also slows the increase in healthcare institutions’ public-welfare strategies. Government and international organizations remain active, but overall convergence efficiency is reduced.

1.
$R_{3}=1.2$ : low cost of noncompliance

Enterprises’ social responsibility probability remains comparatively low and slow to increase, suggesting a weaker impetus to operate responsibly. Consequently, healthcare institutions also slow down their public-welfare efforts. While government and international organizations keep up relatively strong engagement, a low noncompliance cost weakens overall regulatory constraints, impeding system-wide progression toward an ideal equilibrium.

In short, raising $R_{3}$ effectively encourages enterprises to adopt socially responsible strategies, which in turn boosts healthcare institutions’ public-welfare orientation. This synergy helps improve the overall efficiency of emergency response. Policymakers should thus consider increasing the cost of noncompliance to enhance corporate accountability and ensure the fair and efficient allocation of medical resources.

#### 5.2.5 Sensitivity analysis of enterprises’ market trust benefit (T)

**Figure 10:**
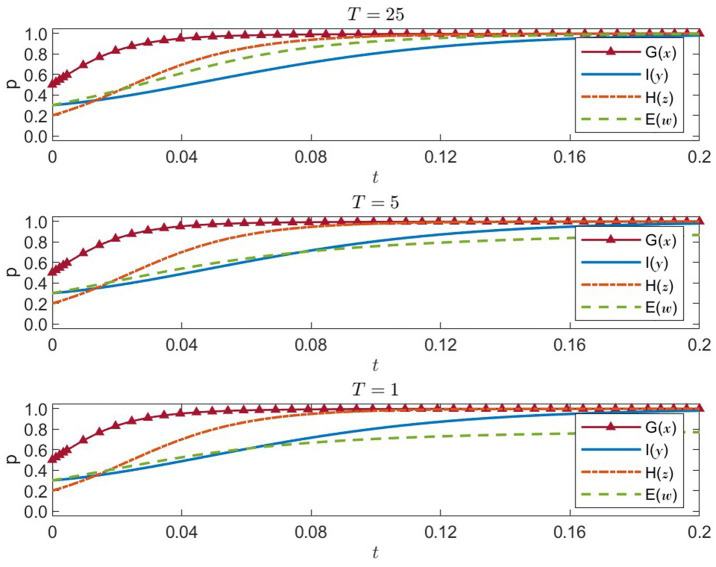
Four-party strategies under enterprise trust benefit T = 25, 5, 1 (x = G, y = I, z = H, w = E).

T denotes the additional market-based trust rewards gained by enterprises that fulfill social responsibilities. We test T={25,5,1} . The results appear in Figure 10.

1.
T=25: high market trust benefit

Enterprises quickly approach a 100% probability of embracing social responsibility, as large trust-related returns strongly incentivize them to behave responsibly. This, in turn, accelerates healthcare institutions’ adoption of public-welfare-first strategies. Both the government and international organizations remain highly proactive, driving rapid convergence to an ideal equilibrium.

1.
T=5: medium market trust benefit

The increase in enterprises’ social responsibility probability moderates, pointing to slightly diminished motivation. Accordingly, healthcare institutions’ movement toward public-welfare strategies slows as well. Government and international organizations continue their positive strategies, but overall convergence to stability takes longer.

1.
T=1: low market trust benefit

Enterprises’ probability of fulfilling social responsibility falls noticeably, rising only slowly, thus revealing insufficient motivation for responsible conduct. Healthcare institutions’ public-welfare probability similarly decreases. Although the government and international organizations remain active, the limited trust-based returns reduce the system’s momentum toward the ideal equilibrium, undermining its overall stability and efficiency.

Overall, enhancing enterprises’ market trust benefit (T) can effectively encourage them to pursue compliance and socially responsible behavior, improving healthcare institutions’ service quality and fostering collaborative efficiency in disaster responses. Governments could employ relevant policies to elevate trust-based rewards for socially responsible firms, thereby improving the overall effectiveness of public health emergency management.

## 6 Discussion

Drawing on a four-party evolutionary game model that incorporates government, international organizations, healthcare institutions, and enterprises, this study probes the mechanisms of collaboration and conflict among multiple stakeholders in natural disaster health emergency response. The findings reveal that strategic evolution among these actors is influenced by a combination of costs and benefits, policy incentives and constraints, and shifts in the external environment. We organize our discussion around four key themes: the “double-edged sword effect” of government regulation, the “time window effect” in international organizational intervention, the “multiplier effect” of healthcare resource efficiency, and the “trust-benefit” mechanism tied to corporate social responsibility. These results both align with and extend earlier research on this topic.

### 6.1 The “double-edged sword effect” of government regulation

The study shows that during disaster emergency response, the government mainly oscillates between two strategies: “Strengthened Regulation” and “Market Deregulation.” When the government strengthens regulation, the immediate result can be considerably heightened efficiency in resource allocation and improved public safety; however, excessive or rigid regulation may dampen market vitality and reduce the ability of social organizations and enterprises to respond independently (51). Conversely, if the government opts for market deregulation, the resulting market dynamics can stimulate innovation and emergency mobilization by enterprises, yet insufficient regulatory oversight or ineffective enforcement can misallocate resources and harm the public interest (52). This is the “double-edged sword effect” of government regulation: on one hand, suitably robust regulation facilitates rapid and efficient emergency response; on the other, overly strong or overly weak regulation can undermine the overall performance of disaster response (6). The simulation results likewise indicate that while enhancing enforcement capacity (e.g., stricter penalties) often boosts the willingness of healthcare institutions and enterprises to act in the public interest, excessively high fines or overly rigid regulatory measures can reduce firms’ motivation and weaken long-term recovery outcomes.

### 6.2 The “time window effect” of international organizational intervention

Rapid response and sustained cooperation from international organizations following a disaster play a pivotal role in strengthening resilience and maintaining health levels in the affected areas. The results suggest that direct intervention from international organizations early in the crisis—through funding and humanitarian assistance—can fill local resource gaps and mitigate social disruption (53). Nevertheless, if such organizations intervene to the point that local actors become overly dependent, they may inadvertently erode local government and organizational capacity for self-recovery and management (54). Thus, the effectiveness of international interventions often hinges on a “time window effect”: while early, short-term direct intervention is crucial for efficient emergency rescue, the intermediate and later phases call for gradual, indirect coordination aimed at developing the self-governance and reconstruction capacities of local governments, healthcare institutions, and enterprises (55). In the simulations, when the “dependency loss” related to international organizations is high but large-scale direct intervention persists, local stakeholders become less enthusiastic about collaborative evolution. This finding resonates with the widely endorsed “relief–recovery–development” pathway in post-disaster humanitarian work.

### 6.3 The “multiplier effect” of healthcare institutions’ resource efficiency

Healthcare institutions shoulder a central role during post-disaster health emergencies, taking on emergency medical care, infectious disease control, resource deployment, and psychological support (56, 57). Research indicates that advance planning and optimal allocation of personnel and equipment enable healthcare institutions to achieve higher resource efficiency and stronger collaborative capabilities once disasters strike (58). Consistent with these ideas, our findings show that when healthcare institutions opt for a “Public Welfare First” strategy and effectively leverage resource support from the government and enterprises, the result can be a “multiplier effect,” wherein modest investments deliver a disproportionately large boost in emergency performance. By contrast, focusing solely on “cost control” or “maximizing efficiency” can cause shortages or insufficient services in emergencies (59). Consequently, public policies must guide healthcare institutions to balance public-welfare priorities with cost-effectiveness. Specifically, initiatives such as fiscal subsidies and price policies can encourage institutions to improve healthcare quality while monitoring expenses; in addition, cross-sectoral collaboration and information-sharing are essential to ensure that the overall healthcare system remains resilient in the face of disasters (60).

### 6.4 The “trust-benefit” mechanism of corporate social responsibility

Enterprises are indispensable in modern disaster relief and reconstruction efforts—not just through funding and material contributions, but also via digital infrastructure, logistics management, and technological innovation (61, 62). The study demonstrates that corporate social responsibility can strengthen societal trust and brand reputation, forming a “trust-benefit” mechanism. Firms that invest more heavily in disaster relief and rebuilding earn positive public and governmental recognition—resulting in reputational and sometimes policy advantages—which over the longer term can bolster financial returns (63). On the other hand, enterprises that pursue only short-term “profit maximization” risk neglecting social and environmental responsibilities and, in so doing, harm affected communities’ well-being and invite possible sanction by regulators or consumer boycotts (64). The simulation shows that when enterprises face higher costs for noncompliance and reap higher trust returns, they become significantly more inclined to fulfill social responsibilities, benefiting both themselves and the public good.

In sum, this research uses a four-party evolutionary game model to analyze how multiple actors collaborate in disaster emergency response. The results indicate that government regulation, international assistance, healthcare institutions’ operational decisions, and corporate social responsibility jointly create a complex, dynamic system for emergency collaboration. Under varying disaster scenarios and time frames, each actor’s strategy shifts in response to changes in costs and benefits and incentives and constraints. In line with prior literature, our study refines our understanding of how to balance strong government oversight with market mechanisms, how to time direct interventions and indirect collaboration from international organizations, how to incentivize healthcare institutions to prioritize public welfare, and how corporate social responsibility aligns with long-term economic interests. These insights inform the design of more effective public policies, suggesting that a well-curated mix of measures—such as regulatory strength, subsidies and rewards, and international coordination policies—can motivate all actors to adopt cooperative strategies that enhance emergency response efficiency and social welfare at different stages of disaster.

Importantly, the evolutionary trajectories derived from our model are not only abstract representations of strategic interactions but also carry direct implications for public health outcomes. For example, when government strategies converge toward strengthened regulation, emergency resources are more effectively allocated, leading to reduced mortality and morbidity in disaster-affected populations. Similarly, enterprises adopting social responsibility and healthcare institutions prioritizing public welfare enhance the resilience of the health system, thereby improving access to timely treatment and reducing long-term public health burdens. Conversely, equilibria dominated by deregulation or cost-control strategies may undermine equity and quality of care, ultimately worsening population health outcomes. This linkage highlights how institutional strategies translate into measurable impacts on disaster-related public health performance. Our findings resonate with recent empirical studies on cross-sectoral coordination in Asia during COVID-19 and natural disasters, which highlight the importance of integrating public, private, and international actors in achieving resilient health governance (65–68). These cases provide further evidence that institutional collaboration, as modeled in our evolutionary game framework, has tangible effects on public health resilience.

## 7 Conclusion

The strategic interactions analyzed in this study have direct implications for measurable public health outcomes. When governments and enterprises converge toward cooperative and welfare-oriented strategies—such as strengthened regulation and corporate social responsibility—resources are allocated more efficiently, emergency response becomes faster, and essential medical services reach affected populations more equitably. These shifts can lead to reductions in disaster-related mortality and morbidity, as well as improved service delivery and recovery effectiveness. Conversely, when strategies lean toward deregulation or profit maximization, coordination efficiency declines, health system responsiveness weakens, and disparities in post-disaster healthcare outcomes are likely to increase. Thus, the evolutionary dynamics identified here provide a theoretical explanation for how institutional decisions translate into real-world public health performance during natural disaster response.

Using evolutionary game theory, this study analyzes the mechanisms of collaborative evolution among governments, international organizations, healthcare institutions, and enterprises in natural disaster emergency response. The findings illustrate the “double-edged sword effect” of government regulation, the “time window effect” of international organizational intervention, the “multiplier effect” of healthcare resource efficiency, and the “trust-benefit” mechanism underlying corporate social responsibility. These insights offer novel approaches for optimizing public policy, showing that well-designed policy instruments can guide all parties toward pursuing strategies that serve the public good—thereby boosting overall emergency efficiency and social welfare. Future work should delve deeper into empirical and case-based studies to enhance the practical relevance and policy effectiveness of such models.

## Data Availability

The original contributions presented in the study are included in the article/Supplementary material, further inquiries can be directed to the corresponding author.
