# Supplementary material for: Natural disaster emergency response from a public policy perspective: a four-party evolutionary game among government, international organizations, healthcare institutions, and enterprises
Source: Front Public Health. 2025 Nov 5;13:1595034. doi: 10.3389/fpubh.2025.1595034 (PMC12626785; doi:10.3389/fpubh.2025.1595034)
Supplement: Supplementary file 1 [file Supplementary_file_1.docx]

**Mathematical Derivations and Stability Analysis for Four-Party Evolutionary Game**

**1 Replicator Dynamic Equation and Equilibrium Points for the Government’s “Strengthened Regulation” Strategy**

Drawing on the payoff matrix for whether the government chooses to strengthen regulation, we calculate the expected payoffs under the different strategy profiles and formulate the corresponding replicator dynamic equation.

- Expected Payoff of Strengthened Regulation, ${Ex}_{1}$：

$${Ex}_{1}=yzw(B_{1}-C_{1})+yz(1-w)(B_{1}-C_{1}+P)+y(1-z)w(B_{1}-C_{1}+P)+y(1-z)(1-w)(B_{1}-C_{1}+P)+(1-y)zw(B_{1}-C_{1})+(1-y)z(1-w)(B_{1}-C_{1}+P)+(1-y)(1-z)w(B_{1}-C_{1}+P)+(1-y)(1-z)(1-w)(B_{1}-C_{1}+P)$$

- Expected Payoff of Market Deregulation, $Ex_{2}:$

$$Ex_{2}=yzw(B_{2}-C_{2})+yz(1-w)(B_{2}-C_{2}-R_{1}-S)+y(1-z)w(B_{2}-C_{2}-R_{1}-S)+y(1-z)(1-w)(B_{2}-C_{2}-R_{1}-S)+(1-y)zw(B_{2}-C_{2})+(1-y)z(1-w)(B_{2}-C_{2}-R_{1}-S)+(1-y)(1-z)w(B_{2}-C_{2}-R_{1}-S)+(1-y)(1-z)(1-w)(B_{2}-C_{2}-R_{1}-S)$$

- Average Expected Payoff for the Government, $Ex$**:**

$$Ex=x{Ex}_{1}+(1-x)Ex_{2}$$

By evolutionary game theory, the replicator dynamic equation for the government’s “Strengthened Regulation” strategy is:

$$F(x)=\frac{dx}{dt}=x({Ex}_{1}-Ex)=x(1-x)(B_{1}-B_{2}-C_{1}+C_{2}+P+R_{1}+S-Pzw-R_{1}zw-Szw)$$

Setting$F(x)=\frac{dx}{dt}=0$ allows us to solve for potential equilibrium points in the evolutionary process:

1. When $w=w^{*}=\frac{B_{1}-B_{2}-C_{1}+C_{2}+P+R_{1}+S}{Pz+R_{1}z-Sz}$,$F(x)\equiv0$. This implies that, along this value of $w$, any $x$ on the $x$-axis yields a stable state in which the government’s strategy does not change over time.
2. When $w\neq\frac{B_{1}-B_{2}-C_{1}+C_{2}+P+R_{1}+S}{Pz+R_{1}z-Sz}$, then$x=0$or $x=1$ are the two possible equilibrium points. By the stability theorem of the replicator dynamic, if $\frac{dF(x)}{dx}<0$ at one of these points, that point is an Evolutionarily Stable Strategy (ESS). Differentiating $F(x)$ with respect to $x$ gives:

$$\frac{dF(x)}{dx}=(1-2x)(B_{1}-B_{2}-C_{1}+C_{2}+P+R_{1}+S-Pzw-R_{1}zw-Szw)$$

- Case i: If $0<w<\frac{B1-B2-C1+C2+P+R1+S}{Pz+R1z-Sz}$, ${\frac{dF(x)}{dx}│}_{x=0}<0,{\frac{dF(x)}{dx}│}_{x=1}>0$. Therefore, $x=0$ is the equilibrium strategy for government behavior, implying that the government tends to adopt “Market Deregulation.”
- Case ii: If $\frac{B1-B2-C1+C2+P+R1+S}{Pz+R1z-Sz}<w<1$, ${\frac{dF(x)}{dx}│}_{x=1}<0,{\frac{dF(x)}{dx}│}_{x=0}>0$. Therefore, $x=1$ is the equilibrium strategy for government behavior, implying that the government tends to adopt “Strengthened Regulation.”

**2 Replicator Dynamic Equation and Equilibrium Points for International Organizations’ “Direct Intervention”**

Using the payoff matrix for international organizations that either directly intervene or indirectly coordinate, we calculate the expected payoffs and derive the replicator dynamic equation.

- Expected Payoff of Direct Intervention,$Ey_{1}$:

$$Ey_{1}=xzw\left( B_{3}-C_{3}+A \right)+xz\left( 1-w \right)\left( B_{3}-C_{3}+A \right)+x\left( 1-z \right)w\left( B_{3}-C_{3}+A \right)+x(1-z)(1-w)(B_{3}-C_{3}+A)+(1-x)zw(B_{3}-C_{3}-D+A)+(1-x)z(1-w)(B_{3}-C_{3}-D+A)+(1-x)(1-z)w(B_{3}-C_{3}-D)+(1-x)(1-z)(1-w)(B_{3}-C_{3}-D)$$

- Expected Payoff of Indirect Coordination, $Ey_{2}$:

$$Ey_{2}=xzw(B_{4}-C_{4}+A)+xz(1-w)(B_{4}-C_{4}+A)+x(1-z)w(B_{4}-C_{4}+A)+x(1-z)(1-w)(B_{4}-C_{4}+A)+(1-x)zw(B_{4}-C_{4}+A)+(1-x)z(1-w)(B_{4}-C_{4}+A)+(1-x)(1-z)w(B_{4}-C_{4})+(1-x)(1-z)(1-w)(B_{4}-C_{4})$$

- Average Expected Payoff for International Organizations, $Ey$:

$$Ey_{1}=yEy_{1}+(1-y)Ey_{2}$$

By evolutionary game theory, the replicator dynamic equation for “Direct Intervention” by international organizations is:

$$F(y)=\frac{dy}{dt}=x(Ey_{1}-Ey)=y(1-y)(B_{3}-B_{4}-C_{3}+C_{4}-D+Dx)$$

Setting$F(y)=\frac{dy}{dt}=0$ allows us to solve for potential equilibrium points in the evolutionary process:

1. When $x=x*=\frac{B_{4}-B_{3}+C_{3}-C_{4}+D}{D}$,$F(y)\equiv0$. This means that for this value of $x$, any $y$ on the $y$-axis remains stable, so the international organization’s strategy does not change over time.
2. When $x\neq\frac{B_{4}-B_{3}+C_{3}-C_{4}+D}{D}$,then$y=0$or $y=1$ are the two possible equilibrium points. By the stability theorem of the replicator dynamic, if $\frac{dF(y)}{dy}<0$ at one of these points, that point is an Evolutionarily Stable Strategy (ESS). Differentiating $F(y)$ with respect to $y$ gives:

$$\frac{dF(y)}{dy}=(1-2y)(B_{3}-B_{4}-C_{3}+C_{4}-D+Dx)$$

- Case i: If $0<x<\frac{B_{4}-B_{3}+C_{3}-C_{4}+D}{D}$, ${\frac{dF(y)}{dy}│}_{y=0}<0,{\frac{dF(y)}{dy}│}_{y=1}>0$. Thus, $y=0$ is the equilibrium, indicating the international organization prefers “Indirect Coordination.”
- Case ii: If $\frac{B_{4}-B_{3}+C_{3}-C_{4}+D}{D}<x<1$, ${\frac{dF(y)}{dy}│}_{y=1}<0,{\frac{dF(y)}{dy}│}_{y=0}>0$. Thus, $y=1$ is the equilibrium, indicating the international organization prefers “Direct Intervention.”

**3 Replicator Dynamic Equation and Equilibrium Points for Healthcare Institutions’ “Public Welfare First” Strategy**

Using the payoff matrix for whether healthcare institutions prioritize public welfare, we calculate the expected payoffs and derive the replicator dynamic equation.

- Expected Payoff of Public Welfare First, $Ez_{1}$:

$$Ez_{1}=xyw(B_{5}-C_{5}+U)+xy(1-w)(B_{5}-C_{5}+U)+x(1-y)w(B_{5}-C_{5}+U)+x(1-y)(1-w)(B_{5}-C_{5}+U)+(1-x)yw(B_{5}-C_{5}+U)+(1-x)y(1-w)(B_{5}-C_{5})+(1-x)(1-y)w(B_{5}-C_{5}+U)+(1-x)(1-y)(1-w)(B_{5}-C_{5})$$

- Expected Payoff of Cost Control, $Ez_{2}$:

$$Ez_{2}=xyw(B_{6}-C_{6})+xy(1-w)(B_{6}-C_{6}-R_{2})+x(1-y)w(B_{6}-C_{6})+x(1-y)(1-w)(B_{6}-C_{6}-R_{2})+(1-x)yw(B_{6}-C_{6}-R_{2})+(1-x)y(1-w)(B_{6}-C_{6}-R_{2})+(1-x)(1-y)w(B_{6}-C_{6}-R_{2})+(1-x)(1-y)(1-w)(B_{6}-C_{6}-R_{2})$$

- Average Expected Payoff for Healthcare Institutions, $Ez$:

$$Ez=zEz_{1}+(1-z)Ez_{2}$$

By evolutionary game theory, the replicator dynamic equation for the “Public Welfare First” strategy is:

$$F(z)=\frac{dz}{dt}=z(Ez_{1}-Ez)=z(1-z)(B_{5}-B_{6}-C_{5}+C_{6}+R_{2}+Uw+Ux-R_{2}wx-Uwx)$$

Setting$F(y)=\frac{dy}{dt}=0$ allows us to solve for potential equilibrium points in the evolutionary process:

1. When $x=x*=\frac{B_{5}-B_{6}-C_{5}+C_{6}+R_{2}+Uw}{R_{2}w+Uw-U}$,$F(z)\equiv0$. This means that along this $x$, any $z$ on the $z$-axis remains stable, implying no change in the healthcare institution’s strategy over time.
2. When $x\neq\frac{B_{5}-B_{6}-C_{5}+C_{6}+R_{2}+Uw}{R_{2}w+Uw-U}$,then$z=0$or $z=1$ are the two possible equilibrium points. By the stability theorem of the replicator dynamic, if $\frac{dF(z)}{dz}<0$ at one of these points, that point is an Evolutionarily Stable Strategy (ESS). Differentiating $F(z)$ with respect to $z$ gives:

$$\frac{dF(z)}{dz}=(1-2z)(B_{5}-B_{6}-C_{5}+C_{6}+R_{2}+Uw+Ux-R_{2}wx-Uwx)$$

- Case i: If $0<x<\frac{B_{5}-B_{6}-C_{5}+C_{6}+R_{2}+Uw}{R_{2}w+Uw-U}$, ${\frac{dF(z)}{dz}│}_{z=0}<0,{\frac{dF(z)}{dz}│}_{z=1}>0$. Thus, $z=0$ is the equilibrium, indicating that healthcare institutions favor “Cost Control.”
- Case ii: If $\frac{B_{5}-B_{6}-C_{5}+C_{6}+R_{2}+Uw}{R_{2}w+Uw-U}<x<1$, ${\frac{dF(z)}{dz}│}_{z=1}<0,{\frac{dF(z)}{dz}│}_{z=0}>0$. Thus, $z=1$ is the equilibrium, indicating that healthcare institutions favor “Public Welfare First.”

**4 Replicator Dynamic Equation and Equilibrium Points for Enterprises’ “Social Responsibility” Strategy**

Using the payoff matrix for whether enterprises fulfill social responsibility, we calculate the expected payoffs and derive the replicator dynamic equation.

- Expected Payoff of Social Responsibility, $Ew_{1}$:

$$Ew_{1}=xyz(B_{7}-C_{7}+T)+xy(1-z)(B_{7}-C_{7})+x(1-y)z(B_{7}-C_{7}+T)+x(1-y)(1-z)(B_{7}-C_{7})+(1-x)yz(B_{7}-C_{7})+(1-x)y(1-z)(B_{7}-C_{7})+(1-x)(1-y)z(B_{7}-C_{7})+(1-x)(1-y)(1-z)(B_{7}-C_{7})$$

- Expected Payoff of Profit Maximization,$Ew_{2}$:

$$Ew_{2}=xyz(B_{8}-C_{8})+xy(1-z)(B_{8}-C_{8}-R_{3})+x(1-y)z(B_{8}-C_{8}-R_{3})+x(1-y)(1-z)(B_{8}-C_{8}-R_{3})+(1-x)yz(B_{8}-C_{8})+(1-x)y(1-z)(B_{8}-C_{8}-R_{3})+(1-x)(1-y)z(B_{8}-C_{8})+(1-x)(1-y)(1-z)(B_{8}-C_{8}-R_{3})$$

- Average Expected Payoff for Enterprises, $Ew$:

$$Ew=wEw_{1}+(1-w)Ew_{2}$$

By evolutionary game theory, the replicator dynamic equation for enterprises’ “Social Responsibility” is:

$$F(w)=\frac{dw}{dt}=w(Ew_{1}-Ew)=w(1-w)(B_{7}-B_{8}-C_{7}+C_{8}+R_{3}-R_{3}z+R_{3}xz+Txz-R_{3}xyz)$$

Setting$F(w)=\frac{dy}{dt}=0$ allows us to solve for potential equilibrium points in the evolutionary process:

1. When $z=z*=\frac{B_{7}-B_{8}-C_{7}+C_{8}+R_{3}}{R_{3}-R_{3}x-Tx+R_{3}xy}$,$F(w)\equiv0$. This means that along this $x$, any $z$ on the $z$-axis remains stable, implying no change in the enterprises’s strategy over time.
2. When $z\neq\frac{B_{7}-B_{8}-C_{7}+C_{8}+R_{3}}{R_{3}-R_{3}x-Tx+R_{3}xy}$,then$w=0$or $w=1$ are the two possible equilibrium points. By the stability theorem of the replicator dynamic, if $\frac{dF(w)}{dw}<0$ at one of these points, that point is an Evolutionarily Stable Strategy (ESS). Differentiating $F(w)$ with respect to $w$ gives:

$$\frac{dF(w)}{dw}=(1-2z)(B_{7}-B_{8}-C_{7}+C_{8}+R_{3}-R_{3}z+R_{3}xz+Txz-R_{3}xyz)$$

- Case i: If $0<z<\frac{B_{7}-B_{8}-C_{7}+C_{8}+R_{3}}{R_{3}-R_{3}x-Tx+R_{3}xy}$, ${\frac{dF(w)}{dw}│}_{w=0}<0,{\frac{dF(w)}{dw}│}_{w=1}>0$. Thus, $w=0$ is the equilibrium, indicating that enterprises lean toward “Profit Maximization.”
- Case ii: If $\frac{B_{7}-B_{8}-C_{7}+C_{8}+R_{3}}{R_{3}-R_{3}x-Tx+R_{3}xy}<z<1$, ${\frac{dF(w)}{dw}│}_{w=1}<0,{\frac{dF(w)}{dw}│}_{w=0}<0$ Thus, $w=1$ is the equilibrium, indicating that enterprises opt for “Social Responsibility.”

**5 Analysis of Equilibrium Strategies in the Evolutionary Game Model**

From the above analysis, we obtain the four-dimensional dynamic system of the four-party evolutionary game:

$$Fx(x,y,z,w)=x(1-x)(B_{1}-B_{2}-C_{1}+C_{2}+P+R_{1}+S-Pzw-R_{1}zw-Szw)$$

$$Fy(x,y,z,w)=y(1-y)(B_{3}-B_{4}-C_{3}+C_{4}-D+Dx)$$

$$Fz(x,y,z,w)=z(1-z)(B_{5}-B_{6}-C_{5}+C_{6}+R_{2}+Uw+Ux-R_{2}wx-Uwx)$$

$$Fw(x,y,z,w)=w(1-w)(B_{5}-B_{6}-C_{5}+C_{6}+R_{2}+Uw+Ux-R_{2}wx-Uwx)$$

Solving $Fx\left( x,y,z,w \right)=0, Fy\left( x,y,z,w \right)=0, Fz\left( x,y,z,w \right)=0, Fw\left( x,y,z,w \right)=0$ yields 16 possible pure-strategy equilibrium points:

$$E_{1}\left( 0,0,0,0 \right),E_{2}\left( 1,0,0,0 \right),E_{3}\left( 0,1,0,0 \right),E_{4}\left( 0,0,1,0 \right),E_{5}\left( 0,0,0,1 \right),E_{6}\left( 1,1,0,0 \right),E_{7}\left( 1,0,1,0 \right),E_{8}\left( 0,1,1,0 \right),$$

$$E_{9}\left( 1,0,0,1 \right),E_{10}\left( 0,1,0,1 \right),E_{11}\left( 0,0,1,1 \right),E_{12}\left( 1,1,1,0 \right),E_{13}\left( 1,1,0,1 \right),E_{14}\left( 1,0,1,1 \right),E_{15}\left( 0,1,1,1 \right),E_{16}\left( 1,1,1,1 \right).$$

The Jacobian matrix of this system is

$J=\left[ \begin{matrix} \begin{matrix} \frac{\partial F_{x}\left( x,y,z,w \right)}{\partial x} & \frac{\partial F_{x}\left( x,y,z,w \right)}{\partial y} \\ \frac{\partial F_{y}\left( x,y,z,w \right)}{\partial x} & \frac{\partial F_{y}\left( x,y,z,w \right)}{\partial y} \end{matrix} & \begin{matrix} \frac{\partial F_{x}\left( x,y,z,w \right)}{\partial z} & \frac{\partial F_{x}\left( x,y,z,w \right)}{\partial w} \\ \frac{\partial F_{y}\left( x,y,z,w \right)}{\partial z} & \frac{\partial F_{y}\left( x,y,z,w \right)}{\partial w} \end{matrix} \\ \begin{matrix} \frac{\partial F_{z}\left( x,y,z,w \right)}{\partial x} & \frac{\partial F_{z}\left( x,y,z,w \right)}{\partial y} \\ \frac{\partial F_{w}\left( x,y,z,w \right)}{\partial x} & \frac{\partial F_{w}\left( x,y,z,w \right)}{\partial y} \end{matrix} & \begin{matrix} \frac{\partial F_{z}\left( x,y,z,w \right)}{\partial z} & \frac{\partial F_{z}\left( x,y,z,w \right)}{\partial w} \\ \frac{\partial F_{w}\left( x,y,z,w \right)}{\partial z} & \frac{\partial F_{w}\left( x,y,z,w \right)}{\partial w} \end{matrix} \end{matrix} \right]$，

whose eigenvalues determine the stability of each of the 16 pure-strategy equilibrium points. If all eigenvalues of $J$evaluated at an equilibrium point are negative, that point is an Evolutionarily Stable Strategy (ESS). If at least one eigenvalue is positive, the point is unstable. **Table 3** summarizes the stability conditions for these 16 pure-strategy equilibria.

According to these conditions, the differences between benefits and costs determine the choices of each of the four stakeholders. Referring to Punctuated Equilibrium Theory (PET), policy typically remains in a stable state over an extended period and undergoes significant changes only under certain conditions—often triggered by major events, shifts in public opinion, or changes in government. In the context of disaster emergency response, policy might evolve from an initial stable state to experiencing major readjustments at later stages. We divide the strategy evolution of natural disaster health emergency response into four phases—Stable Preparedness, Immediate Response, Adaptive Adjustment, Recovery and Reconstruction—and analyze the stability of equilibrium points in each phase.

**5.1 Stable Preparedness Phase**

Before a disaster occurs, the system is in a long-term stable state. Here, the government tends to choose “Strengthened Regulation” to maintain social order and efficiently allocate resources, ensuring robust preparation for potential disasters. International organizations do not intervene, allowing the government sufficient autonomy to develop its own disaster prevention and preparedness measures. Healthcare institutions do not prioritize public welfare, focusing on operational stability and service quality to ensure effective medical support when a disaster strikes. Enterprises do not fulfill social responsibility, concentrating on profit maximization and operational stability so that basic economic functions are maintained in case of a disaster. This phase corresponds to the equilibrium point $E_{2}\left( 1,0,0,0 \right).$

According to Table 3, for $E_{2}$​ to be stable, the following four conditions must hold:

1. $B_{2}-C_{2}-R_{1}<B_{1}-C_{1}+P-S$: If the net payoff from “Market Deregulation” （$B_{2}-C_{2}-R_{1}$）is lower than the net payoff from “Strengthened Regulation” ($B_{1}-C_{1}+P-S$), the government leans toward “Strengthened Regulation.”
2. ${B_{3}-C_{3}<B}_{4}-C_{4}$: If the net payoff of “Direct Intervention” ($B_{4}-C_{4}$) is less than that of “Indirect Coordination” ($B_{3}-C_{3}$​), international organizations favor “Indirect Coordination.”
3. $B_{5}-C_{5}+U<B_{6}-C_{6}-R_{2}$: If the net payoff of “Public Welfare First” ($B_{5}-C_{5}+U$) is lower than that of “Cost Control” ($B_{6}-C_{6}-R_{2}$​), healthcare institutions favor “Cost Control.”
4. $B_{7}-C_{7}<B_{8}-C_{8}-R_{3}$: If the net payoff of “Social Responsibility” ($B_{7}-C_{7}$​) is lower than that of “Profit Maximization” ($B_{8}-C_{8}-R_{3}$​), enterprises favor “Profit Maximization.”

During this phase, the government strengthens regulation and planning for disaster preparedness; healthcare institutions refine internal processes to enhance operational efficiency; enterprises may invest in R&D to improve their risk-resilience. Because international organizations do not intervene, the government develops and implements disaster prevention and emergency plans autonomously.

**5.2 Immediate Response Phase**

Once a disaster occurs, stakeholders rapidly adjust their strategies to meet urgent needs. The government continues with “Strengthened Regulation” to ensure the swift allocation of resources and maintenance of social order—critical in the disaster’s early stages. International organizations move to “Direct Intervention” by supplying urgent material, financial, and humanitarian aid to alleviate initial impacts. Healthcare institutions do not prioritize public welfare but focus on emergency medical services to guarantee that victims receive basic care. Enterprises refrain from social responsibility and concentrate on maintaining operational stability. This corresponds to equilibrium $E_{6}\left( 1,1,0,0 \right).$

Table 3 shows that the following four conditions must hold for $E_{6}$​ to be stable:

1. $B_{2}-C_{2}-R_{1}<B_{1}-C_{1}+P-S$: Governments again lean toward “Strengthened Regulation” if the net payoff from deregulation is lower than that from regulation.
2. $B_{4}-C_{4}<B_{3}-C_{3}$: International organizations prefer “Direct Intervention” if the net payoff from “Indirect Coordination” is lower than from direct aid.
3. $B_{5}-C_{5}+U<B_{6}-C_{6}-R_{2}$: Healthcare institutions focus on “Cost Control” if its net payoff exceeds that of “Public Welfare First.”
4. $B_{7}-C_{7}<B_{8}-C_{8}-R_{3}$: Enterprises favor “Profit Maximization” if its net payoff is higher than that of “Social Responsibility.”

During this phase, the government rapidly deploys resources, while international organizations provide immediate assistance. Healthcare institutions concentrate on emergency medical treatment, and enterprises can support relief efforts through donations or volunteer services. Government–international organization cooperation is crucial to ensure orderly resource distribution and social stability.

**5.3 Adaptive Adjustment Phase**

As disaster response progresses, stakeholders adapt to the evolving conditions, adjusting their strategies to optimize resource allocation and emergency effectiveness. The government continues “Strengthened Regulation,” ensuring effective resource distribution and social order. International organizations persist with “Direct Intervention,” offering necessary ongoing assistance. Healthcare institutions begin to prioritize “Public Welfare First,” thus improving service quality and addressing disaster-related health challenges. Emphasizing public welfare enhances the equity and quality of medical care for affected populations. Enterprises remain focused on “Profit Maximization” to maintain post-disaster economic viability. This phase corresponds to equilibrium $E_{12}\left( 1,1,1,0 \right)$.

Table 3 indicates four conditions for $E_{12}$​ to be stable:

1. $B_{2}-C_{2}-R_{1}<B_{1}-C_{1}+P-S$: Governments prefer regulation over deregulation if the latter’s net payoff is lower.
2. $B_{4}-C_{4}<B_{3}-C_{3}$: International organizations favor “Direct Intervention” if that yields higher net payoff than “Indirect Coordination.”
3. ${B_{6}-C_{6}-R_{2}<B}_{5}-C_{5}+U$: Healthcare institutions focus on “Cost Control” if its net payoff exceeds that of “Public Welfare First.”
4. $B_{7}-C_{7}<B_{8}-C_{8}-R_{3}$: Enterprises opt for “Profit Maximization” if the net payoff of social responsibility is lower than pursuing profit.

In this phase, healthcare institutions should refine internal processes to deliver higher-quality care, and government policies can incentivize these institutions to embrace public welfare. International organizations continue their assistance for long-term recovery, while enterprises still prioritize near-term profit but may start exploring ways to contribute to reconstruction without undermining their bottom line.

**5.4 Recovery and Reconstruction Phase**

After the immediate response transitions to a long-term recovery phase, the government maintains “Strengthened Regulation” to ensure effective resource allocation and social stability. International organizations continue providing “Direct Intervention,” offering technical and financial support to help affected regions achieve sustainable recovery. Healthcare institutions prioritize “Public Welfare First” to safeguard public health and deliver high-quality services. Enterprises now shift toward “Social Responsibility,” contributing to the ongoing recovery and rebuilding process for the long haul. This phase corresponds to the equilibrium point $E_{16}\left( 1,1,1,1 \right)$.

Table 3 shows four conditions for $E_{16}$​ to be stable:

1. $B_{2}-C_{2}<B_{1}-C_{1}$: The government favors regulation when the net payoff of deregulation is lower than that of regulation.
2. $B_{4}-C_{4}<B_{3}-C_{3}$: International organizations prefer “Direct Intervention” when its net payoff exceeds that of “Indirect Coordination.”
3. ${B_{6}-C_{6}-R_{2}<B}_{5}-C_{5}+U$: Healthcare institutions favor “Public Welfare First” when it yields higher net benefits than “Cost Control.”
4. ${B_{8}-C_{8}-R_{3}<B}_{7}-C_{7}+T$: Enterprises opt for “Social Responsibility” if it brings greater net benefits than “Profit Maximization.”

This phase represents the ideal state of the system. Each stakeholder’s strategic choice supports a sustainable long-term recovery, helping to maintain social stability. The government formulates extended recovery plans; international organizations continue to provide technical guidance and financial support; healthcare institutions fortify public health infrastructure; and enterprises invest in reconstruction, fulfill their social responsibilities, and help revitalize the affected economy.
